# A review of evaluation approaches for explainable AI with applications in cardiology

**DOI:** 10.1007/s10462-024-10852-w

**Published:** 2024-08-09

**Authors:** Ahmed M. Salih, Ilaria Boscolo Galazzo, Polyxeni Gkontra, Elisa Rauseo, Aaron Mark Lee, Karim Lekadir, Petia Radeva, Steffen E. Petersen, Gloria Menegaz

**Affiliations:** 1 William Harvey Research Institute, NIHR Barts Biomedical Research Centre, Queen Mary University of London, Charterhouse Square, London, EC1M 6BQ UK; 2 Department of Population Health Sciences, University of Leicester, University Rd, Leicester, LE1 7RH UK; 3 Department of Computer Science, University of Zakho, Duhok road, Zakho, Kurdistan Iraq; 4 Department of Engineering for Innovative Medicine, University of Verona, S. Francesco, 22, 37129 Verona, Italy; 5 Artificial Intelligence in Medicine Lab (BCN-AIM), Departament de Matemàtiques i Informàtica, Universitat de Barcelona, Gran Via de les Corts Catalanes, 585, 08007 Barcelona, Spain; 6 Institució Catalana de Recerca i Estudis Avançats (ICREA), Passeig Lluís Companys 23, Barcelona, Spain; 7 Departament de Matemàtiques i Informàtica, Universitat de Barcelona, Gran Via de les Corts Catalanes, 585, 08007 Barcelona, Spain; 8 Barts Heart Centre, St Bartholomew’s Hospital, Barts Health NHS Trust, West Smithfield, London, UK; 9 Health Data Research, London, UK; 10 Alan Turing Institute, London, UK

**Keywords:** Cardiac, AI, XAI, Evaluation

## Abstract

Explainable artificial intelligence (XAI) elucidates the decision-making process of complex AI models and is important in building trust in model predictions. XAI explanations themselves require evaluation as to accuracy and reasonableness and in the context of use of the underlying AI model. This review details the evaluation of XAI in cardiac AI applications and has found that, of the studies examined, 37% evaluated XAI quality using literature results, 11% used clinicians as domain-experts, 11% used proxies or statistical analysis, with the remaining 43% not assessing the XAI used at all. We aim to inspire additional studies within healthcare, urging researchers not only to apply XAI methods but to systematically assess the resulting explanations, as a step towards developing trustworthy and safe models.

**Supplementary Information:**

The online version contains supplementary material available at 10.1007/s10462-024-10852-w.

## Introduction

Cardiovascular diseases are the leading global cause of death and represent a major healthcare burden (Vaduganathan et al. 2022). Advanced artificial intelligence (AI) models, especially those based on deep learning, have shown success in cardiac-related applications (Karatzia et al. 2022), taking advantage of the increasing availability of multi-source data including cardiac imaging techniques (e.g., cardiac magnetic resonance imaging [CMR], X-ray, ultrasound, echocardiograms), electrocardiogram (ECG) and electronic health records (EHR). However, models based on as convolutional neural networks (CNN) and reinforcement learning (e.g., Markov Decision Process and Q-learning) are generally considered black box, especially when it comes to more clinically-oriented applications, as the internal mechanisms and the rationale behind model outputs are not explicit (Linardatos et al. 2020). It is thus difficult for clinicians to trust model predictions which cannot be interpreted and lack transparency (Linardatos et al. 2020; Loh et al. 2022). Accordingly, eXplainable AI (XAI) has been proposed as a possible solution to make AI models more transparent and comprehensible (Mohseni et al. 2021), and thereby to enhance understanding, increase trust, and uncover potential risks associated with complex models (Szabo et al. 2022). In addition, XAI has a potential use in detecting biases in the underlying AI models, leading to improved generalizability and performance. XAI has experienced significant growth over the last few years with several methods being proposed to deal with the peculiarities of the different AI models and data, and providing either local or global explanations (Selvaraju et al. 2017; Chattopadhay et al. 2018; Lundberg and Lee 2017; Ribeiro et al. 2016; Plumb et al. 2018).

At the same time, the quickly growing and changing field of XAI has posed new challenges in the healthcare area, including the necessity of objective evaluations of the resulting explanations (Chaddad et al. 2023). While evaluation methods are often grouped according to different criteria in the literature, a common way of classifying XAI approaches is according to whether user involvement is required (human-centred) or not (computational-centred) (Doshi-Velez and Kim 2017). In particular, three main kinds of evaluations have been proposed:1) human-grounded, 2) application-grounded, 3) functionally-grounded evaluation (Doshi-Velez and Kim 2017). *Human-grounded evaluation* indicates that the XAI explanation is assessed by lay persons, for example by selecting the most reasonable option included in specific questionnaires listing the outcomes of multiple XAI methods. Such approaches might be useful only for simple tasks and can only provide a general sense as to the validity of the explanation. *Application-grounded evaluation* is still human-centred but, in this case, it refers to assessments done by the experts in the specific domain, for example cardiologists in cardiac-related applications. Finally, *functionally-grounded evaluation* indicates that the outcome of XAI is evaluated solely by some kind of proxies, statistical methods or formal definitions of interpretability with no human intervention (computer-centred) (Doshi-Velez and Kim 2017).

More recently, other evaluation approaches are emerging, although these are not included in the current taxonomy. The first one can be referred to as *literature-grounded evaluation*, where the outcome of the XAI is assessed based on comparisons with what is known in the literature and with previous findings. The second one, *guideline-grounded evaluation*, requires following specific guidelines to assess the outcome of XAI. It might involve both application- and functionality-grounded evaluations where the outcomes are evaluated by experts in the domain relying on some kind of proxies.

Starting from this scenario, this review contributes to the body of knowledge of XAI evaluation approaches, methods and metrics focusing on cardiac studies. We commence with an introduction to XAI and provide the taxonomy and the main approaches for evaluating XAI outputs. We then focus on summary statistics derived from a comprehensive literature review of XAI evaluation methods in the cardiac domain, subsequently delving into the practical applications of these XAI evaluation techniques within cardiac research. Lastly, we discuss open issues and future directions.

## Rationale XAI

Arguably, XAI should narrow the gap between model accuracy and transparency by converting *black box* but accurate AI models into a more understandable form. XAI helps to elucidate how a model reached a specific decision, the extent to which model is certain and what are the regions of an image or group of features that dominated the model decision.

Explainability and interpretability are often used interchangeably, which might confuse the reader about what they represent. To clarify their meanings, Table 1 provides their definitions along with those of other common terms used in XAI and generally in AI field.

**Table 1:** Common terminologies in XAI with their definition

Term	Definition
Explainability	Refer to the ability of understanding the internal mechanism and the behavior of a system and to explain why a specific action was made (Salih et al. 2023b)
Interpretability	Reflects the extent to which degree that the model’s output is understandable from human prospective (Salih et al. 2023b)
Transparency	Opposite to black box and has the potential to be understandable by itself (Linardatos et al. 2020)
Transferability	Understanding an AI system in a way can be extended or transferred into another domain and problem (Arrieta et al. 2020)
Trustworthy	That the system is transparent, safe and the output can be trusted (Arrieta et al. 2020)
Fidelity	To what extend does the explanation represent and capture the workings of the AI system? (Lopes et al. 2022)
Fairness	The AI system’s decisions do not exhibit prejudice against any group or individual based on inherent characteristics (Linardatos et al. 2020)
Accountability	The assurance that the AI system can be trusted and works as was presented (Novelli et al. 2023)

**Fig. 1:**
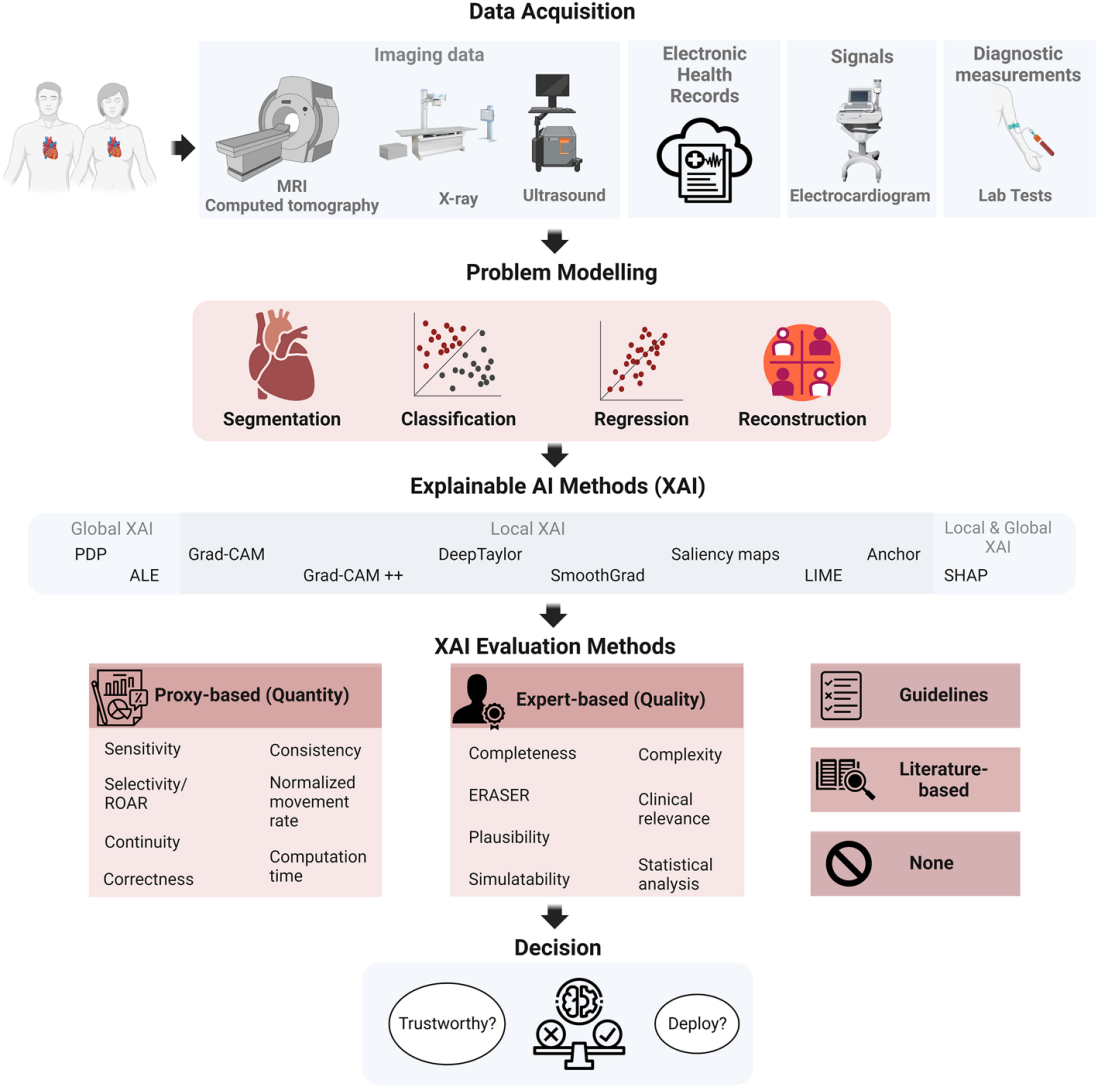
General illustration. *MRI*:magnetic resonance imaging, *PDP* partial dependence plot, *ALE* accumulated local effects, *Grad-CAM* gradient-weighted class activation mapping, *LIME* local interpretable model-agnostic explanations, *SHAP* shapley additive explanations, *ROAR* RemOve And Retrain, *ERASER* evaluating rationales and simple English reasoning. Created with BioRender.com

Figure 1 provides of an overview of the general workflow for an efficient XAI analysis pipeline, designed, in this case, for cardiac AI applications, although readily generalizable with respect to data acquisition methods, model architecture, application of XAI methods, evaluation of XAI outcomes and final decision. When acquiring data for cardiac assessments, the selection of data modalities depends on the aim of the task at hand, including the target disease, but also on other parameters such as cost, resource availability, and time constraints.

The main data types include imaging (e.g., CMR, echocardiography, ultrasound, nuclear perfusion scans) to evaluate the structure and function of the cardiac, ECG for the assessment of the cardiac electric activity, diagnostic measurements from laboratory exams such as blood tests, and other structured and unstructured patient information from EHR (e.g., demographics, risk factors, medical history, clinical notes among others). Notably, some diagnostic measurements, signal data, images and image-derived information may also be present in the patient’s EHR, which, in this context, encompasses all other pertinent patient-related data. Nonetheless, in this review, the term “EHR data” excludes imaging and signal.

According to the specific research or clinical questions, different modelling strategies using machine learning can be designed and developed. More precisely, regression models can be used to predict a continuous variable such as cardiac age, stroke volume or cardiac function parameters, while classification models can be employed to distinguish between two cases (e.g., control vs heart failure). In addition, segmentation models can be used to segment the anatomy of the cardiac and extract CMR metrics, and reconstruction models can help to improve the quality of cardiac images.

Once the optimal model is defined and its performance carefully evaluated (e.g., cross validation, independent test set), XAI methods can be applied to explain and interpret the model. The most appropriate XAI method can be chosen based on the model and data types. For example, SHAP (Shapley Additive Explanations, an XAI based on game theory) can be applied to both imaging and tabular data, while Gradient-weighted Class Activation Mapping (Grad-CAM) and DeepTaylor can be implemented on imaging and signal (e.g., ECG) data. Once a given XAI method has been applied, it is important to evaluate the explanation it provides (although this step is still rarely applied in the current literature and most of the cardiac studies do not focus on this additional analysis). The final step is to evaluate whether to trust, generalize and deploy the XAI after it has been appropriately evaluated.

### Taxonomy of XAI

XAI approaches are typically categorized as either “ante-hoc” or “post-hoc” methods (Salih et al. 2023b). *Ante-hoc* means that the explanation is intrinsic, and the model is self-explanatory (white-box model). On the contrary, *post-hoc* methods require the application of another model to explain the results of the AI model. Linear regression models are examples of *ante-hoc* XAI methods that are simple and directly interpretable. Indeed, the regression coefficients can indicate the importance of the different predictors and how they affect the models. On the other hand, CNN models belong to the *post-hoc* category as they require the application of other models for interpretation.

Another criterion that can be applied to classify a given XAI method is whether it is local or global. *Local* indicates that the resulting explanation can be provided for a specific data point or instance in the model. On the other hand, *global* provides general explanations for all instances in the model, for example the impact of a specific feature in the model for all instances. Grad-CAM (Selvaraju et al. 2017), DeepTaylor (Montavon et al. 2017), Layer-Wise Relevance Propagation (Bach et al. 2015; Wagner et al. 2024), LIME (Ribeiro et al. 2016) and guided backpropagation (Springenberg et al. 2014) are examples of XAI methods that provide local explanations, while partial dependence plots (PDP) (Greenwell et al. 2018), accumulated local effects plots (ALE) (Apley and Zhu 2020) and SHAP (Lundberg and Lee 2017) are examples of XAI models that provide global explanation (though SHAP can provide both kinds of explanation).

In addition, XAI can be categorized into model-specific or model-agnostic. *Model-specific* refers to any XAI model that was developed for a specific machine learning (ML) model. Conversely, *model-agnostic* includes all XAI methods that can be applied to any model, regardless its complexity or simplicity. XAI methods including SHAP and LIME can be considered as *model-agnostic* because they can be applied to any model.

Despite many XAI methods have been developed in the past five years, little attention has been given to the evaluation part and there is no standard measure or metric to assess their outcome yet (Silva et al. 2023). Moreover, XAI methods often assume that the end users in any domain have a certain level of expertise which qualifies them to understand and evaluate the quality and correctness of its outcome. However, such assumption cannot be met in several cases, making difficult a fair assessment of the XAI outcome by the end users (Bruijn et al. 2022). Another concern related to the current XAI methods is the lack of causality in the outcome. More precisely, current AI models primarily rely on identifying associations between the input and the output, which might not necessarily imply causation. Consequently, the explanations generated by XAI methods may not accurately reflect causal association (Molnar et al. 2022; Chou et al. 2022). In addition, current XAI methods based on input perturbations lack robustness against adversarial attacks and can be fooled to produce biased results (Slack et al. 2020).

All points mentioned above will be better illustrated and detailed in the following sections.

## XAI evaluation methods

In this section, we introduce the main XAI evaluation methods, following the current taxonomy and further complementing it with other approaches that we retrieved from the current studies, and we believe being relevant. As introduced in Sect. 1, evaluation methods can be categorized as follows: human-grounded (lay person), application-grounded (expert in the domain), functionality-grounded (proxy), literature-grounded and guideline-grounded. The main examples for each category will be discussed, although for more details on each metric and method we refer the interested readers to specific reviews on this topic as it is out of the scope of the current review (Mohseni et al. 2021; Kumarakulasinghe et al. 2020; Linardatos et al. 2020; Lopes et al. 2022).

### Human and application-grounded evaluations

The approaches belonging to these categories require the participation of humans in the evaluation, either lay persons (human-grounded) or domain experts (application-grounded). Here, the main challenge is that the evaluations done by humans, especially when involving lay persons, are partially subjective, as they depend on the level of expertise, main domain knowledge and individual judgment. Indeed, the same explanation can be satisfying for one user but totally incomprehensible for another and there might be a lack of consensus between participants. However, the involvement of experts in the field might partially mitigate this intrinsic limitation, thus making application-grounded evaluations more suitable especially in the healthcare domain.

In this case, qualitative measures informing on the clinical relevance, plausibility and complexity of a given XAI explanation are usually provided by the experts. The following criteria represent some of the proposed notions to qualitatively evaluate the XAI outcomes.

1. **Completeness:** It can be defined as whether the explanation is complete to the end users or not. Completeness involves full details related to the boundary of the used data, the model, the XAI method, limitations, evaluation metrics and how to interpret the results (Cui et al. 2019).
2. **Simplicity:** It is related to the cases where the task is well-known and related to daily-life issues where it is easy to distinguish and decide if the explanation is good or bad (Montavon et al. 2018).
3. **Evaluating Rationales And Simple English Reasoning (ERASER):** It is a benchmark to evaluate models applied to natural language processing applications. They proposed several metrics to evaluate the explanation considering human rationales as ground truth (DeYoung et al. 2019).
4. **Plausibility:** It is one of the most precious metrics to evaluate any XAI method. It measures if the explanation provided by the machine is inline with the expert explanation and expectation. In other words, it assesses whether a human is convinced by the explanation or not (Jin et al. 2023b).
5. **Simulatability:** It indicates that the model behavior can be predicted when it is applied to new data (Hase and Bansal 2020). This is a significant metric as it means that the end users understand how the simulatable models work. It is divided into two tasks, the first one refers to the user ability to predict the explanation for a given input, while the second one is the ability of the users to predict the changes in the explanation when a given perturbation is applied to the input data.
6. **Complexity:** It indicates the degree of complexity of the explanation when debugging the XAI method. In simple words, it is the needed time to understand the explanation (Cui et al. 2019). In addition, this measure refers to the amount of information held in the XAI outcomes (Gilpin et al. 2018) and is a measure of conciseness, meaning that an explanation should consist of a few strong features (Chalasani et al. 2020), making the interpretation of the XAI outcomes easier and more robust.
7. **Clinical relevance:** It means that the explanation should be in agreement with the physicians’s opinions and support their clinical decision and reasoning (Di Martino and Delmastro 2022). Some proposed frameworks tried to further quantify clinical relevance by calculating additional measures such as the percentage of explanations that are accepted by physicians or the percentage of overlap between XAI and physicians explanation (Kumarakulasinghe et al. 2020).

Another possibility is to combine the evaluations by the experts with statistical analyses (proper of functionally-grounded evaluations) to identify whether there is an agreement between what was depicted by a given XAI method as most relevant (e.g., specific feature or imaging region) and the opinion by the expert. In this way, an objective quantification of the level of concordance can be derived and used as additional metric to evaluate the XAI outcomes.

Importantly, some limitations have to be acknowledged when relying on application-grounded evaluation. Indeed, such an approach is expensive as each study in a specific domain needs its own experts for the assessment, is time consuming, and thus might be less appropriate in critical clinical settings where immediate XAI evaluations are needed (e.g, intensive care units), and might require the involvement of more expert users when the task is particularly demanding. Moreover, for some measures such as complexity and completeness, the partial subjectiveness might still exist despite the involvement of experts, as end users with different level of expertise might lead to different opinions on these metrics.

### Proxy-grounded evaluation

Functionality-grounded (or proxy-grounded) approaches represent methods that use quantitative proxies, metrics, axioms, and statistics to assess the quality of the XAI outcomes. In addition, they might use some formal definitions of explainability or interpretability to evaluate the results. Such methods are promising because they do not require human intervention or experts in the domain, and they can be applied to assess the value and robustness of novel XAI methods (Doshi-Velez and Kim 2017). However, some limitations must be acknowledged also in this case. Firstly, it is hard to determine which is the most suitable proxy to evaluate a given XAI method. Then, this approach does not consider clinical relevance and plausibility, as it does not involve experts. In addition, such methods might be biased by part of the data or by the adopted XAI model, making the evaluations less reliable.

In what follows, we will discuss some of the most common proxies that have been proposed so far for evaluating XAI outcomes.

1. **Sensitivity:** It indicates that if two identical models have different outputs and same input but differ in one feature, then the attribution of that feature should not be zero (Hooker et al. 2019; Sundararajan et al. 2017). In addition, if a feature does not contribute to the model output, then zero attribution should be given to that feature;
2. **Selectivity or RemOve And Retrain (ROAR):** It was proposed to measure the accuracy of attribution estimates in deep neural networks. It evaluates the changes in accuracy a given model experiences when the top features identified by XAI are removed. If a sharp reduction occurs, it is likely that the removed inputs are highly informative and that the XAI importance estimates are correct. If not, this means that the removed features hold only marginal information and thus the XAI outcomes can be considered of poor quality (Hooker et al. 2019; Montavon et al. 2018);
3. **Continuity:** It means that the explanation of two instances should be nearly equivalent if their data points are also nearly equivalent (Montavon et al. 2018). In other words, it is the variation in the explanation in the input domain;
4. **Correctness:** It means that the explanation should correctly explain and identify the main components of the model that mostly drive the outcome (Kuppa and Le-Khac 2020). However, such assumption is hard to define due to the lack of ground truth. In Yalcin et al. (2021), authors defined correctness by building datasets with known explanation and then correlated the explanation with the model accuracy;
5. **Consistency:** It refers to what degree or extent the explanation will be different when different models are applied to the same data (Leventi-Peetz and Weber 2022). In addition, it measures how the explanation will be changed if the input data are altered or transformed compared to the explanation of the original input data (Kuppa and Le-Khac 2020).
6. **Normalized movement rate (NMR):** It was proposed as a measure to assess whether the XAI models are robust against the collinearity among the used predictors in the model (Salih et al. 2022). NMR is calculated by checking and quantifying how the predictors change their indexes in the list of the most informative predictors (from a given XAI method) when the top one is removed iteratively. The smaller the NMR value, the more robust the model against collinearity or the predictors are independent which consequently provide more reliable explanation. On the other hand, the closer the NMR value is to 1, the weaker the model against the collinearity and the explanation is not realistic.
7. **Computation time:** It is another criterion to be considered in the evaluation of the XAI outcomes. It is vital that the required time for generating an explanation is as short as possible, especially in some cases where time is very critical (Kakogeorgiou and Karantzalos 2021). Explainability methods requiring long computation times might be difficult to integrate in complex pipelines when real-time performance is required. However, the trade-off between computational time and accuracy/reliability of the explanations should be always considered, especially in the healthcare domain.

### Literature-grounded evaluation

Besides human-centered and computer-centered approaches, XAI outcomes are often evaluated by the different researchers and users using previous literature findings as benchmarks (*literature-grounded* evaluation). This category of evaluation methods is somehow close to the expert-grounded evaluation as it considers the findings from the experts in the domain. However, this approach has some drawbacks, especially in terms of subjectiveness. Indeed, the users might tend to be more selective while searching in the literature, ending up in choosing the findings that are more in line with their XAI outcomes and partially ignoring the mismatched ones. This might limit the generalizability of the XAI outcomes and might provide only a partial evaluation. While the importance of referring to the state-of-the-art to aid in evaluating a given explanation is undeniable and should be increasingly pursued in all XAI research studies in the healthcare domain, we believe that literature findings should only be used as additional confirmation to prove the reliability and plausibility of the results, and that they should be complemented with other measures and comparisons. Moreover, any different data, model or XAI method should be acknowledged, as these can have a significant impact on the outcomes and subsequent evaluation.

### Guideline-grounded evaluation

Recently, another approach has been proposed to assess the quality of the XAI outcome by relying on guidelines combining both proxy and expert-grounded methods (Chen et al. 2022; Jin et al. 2023a). *Guideline-grounded* evaluation usually assesses the outcome of XAI through a pipeline where the input is given by the XAI outcome and there is a specific evaluation criterion in every step. Seven guidelines’ steps of assessment were proposed by Jin and colleagues (Jin et al. 2023a) to examine any XAI method and its explanations in clinical settings. Such clinical guidelines are mixing both proxy and expert methods including clinical relevance, computational efficiency, informative plausibility, truthfulness, and understandability (Jin et al. 2023b). Another set of guidelines for medical image analysis applications were proposed by Chen et al. (2022), emerging as result of their systematic review paper on 68 studies. The proposed guideline (INTRPRT) has several parts including incorporation (IN), interpretability (IN), target (T), reporting (R), prior (PR), and task (T). The proposed INTRPRT guideline suggests a human-centered design to develop transparent AI in healthcare. More in detail, incorporation indicates including an adequate number of end-users (clinicians) to collaborate with the designers during the construction and assessment of the model. Interpretability refers to the technical aspects of the model to make the model transparent. Target determines the final users of the transparent AI algorithms. Reporting indicates summarizing all approaches and aspects used to evaluate the transparency of the model. Prior in particular points to previous findings, sources or information related to the target users. This will help the designers to understand the end-users better while designing a transparent model. Finally, task refers to the aim of the model, whether it is for segmentation, classification of prediction.

While being promising, such an approach still poses several challenges, given by the complexity in defining general and appropriate guidelines. The different applications in the medical domain might require more faceted and human-centered approaches that should increasingly involve the target end users to build together more transparent models and verify that the assumptions are valid.

## Literature review in numbers

In the current work, we investigated the evaluation methods applied to XAI outcomes in cardiac studies within the existing literature. Following the Preferred Reporting Items for Systematic Reviews and Meta-Analyses (PRISMA) guidelines, we conducted a search across four academic databases, namely Web of Science, Scopus, PubMed, and IEEE Xplore. The aim was to collect all published papers that applied XAI methods in any context related to cardiac applications. It should be noted that the search was limited to papers published in English language, without year restrictions. The search query consisted of four parts: (1) “cardiac” or “heart”, (2) terms related to cardiac imaging acquisition methods or cardiac data, 3) terms related to artificial intelligence methods and a wide range of XAI methods (Table S1). The search encompassed both paper titles and abstracts. The most recent search was conducted on 20/08/2023.

**Fig. 2:**
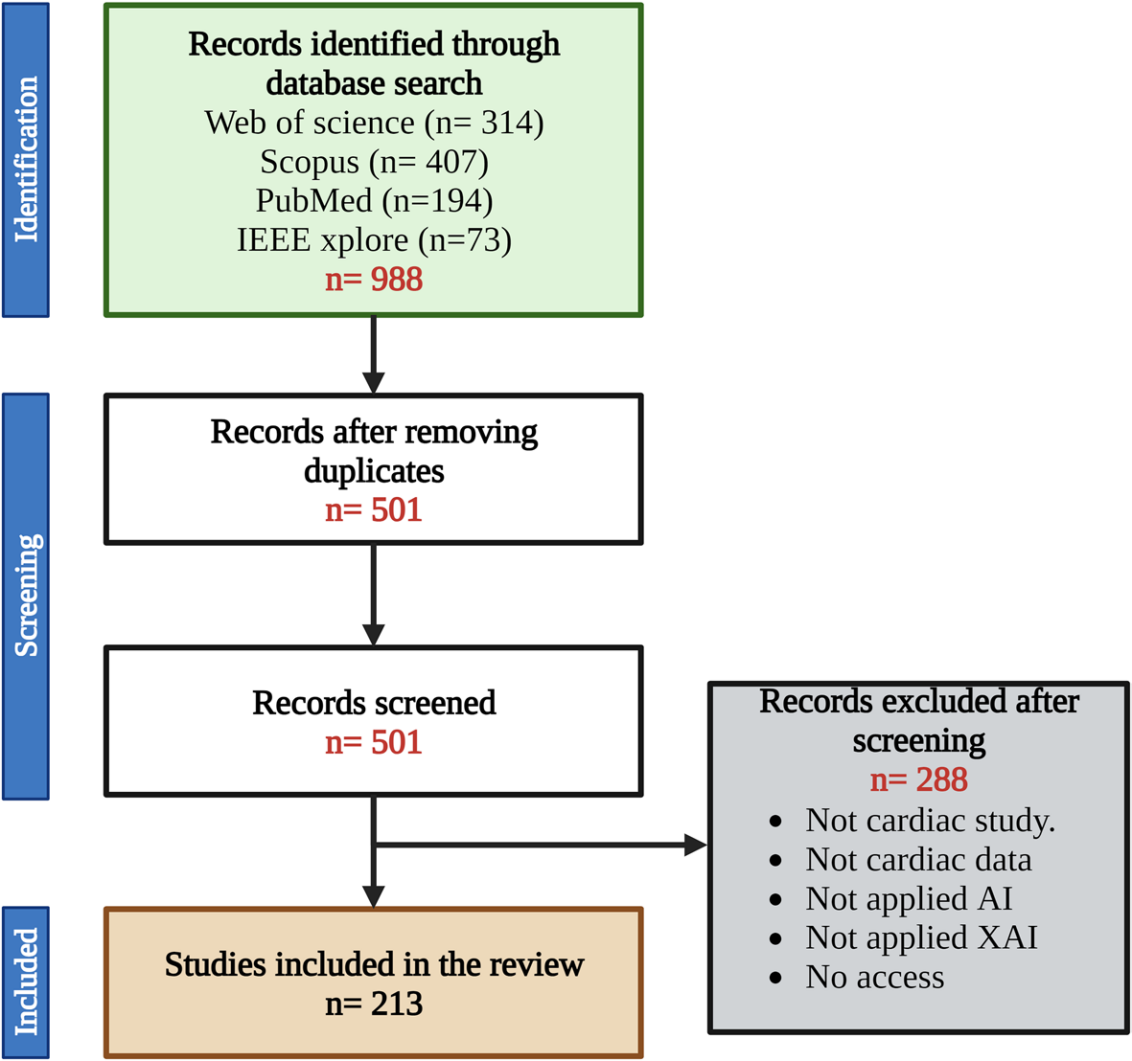
Workflow adhering to PRISMA guidelines, detailing the exclusion and inclusion criterion used in the search process, along with the final number of papers considered in the review

Figure 2 outlines the workflow that was followed to select the studies to include in the review according to the PRISMA guidelines. Following the initial literature search across the four repositories and subsequent removal of duplicate papers, 501 unique papers were collected. Thereafter, these papers were manually assessed to ensure alignment with the aim of this study, excluding those that did not use XAI methods or cardiac data. These steps resulted in a final sample of 213 papers to be included in the review.

Notably, most of the reviewed studies applied classification models (170) to predict a condition versus a control, and they were primarily focused on certain cardiac conditions like arrhythmia’s and ischemic heart disease (IHD). Twenty-three studies used regression models to predict a continuous variable, mainly targeting cardiac age and CMR metrics including left ventricular geometry and left ventricular ejection fraction. Few papers involved segmentation, clustering or image re-construction models in their studies.

**Fig. 3:**
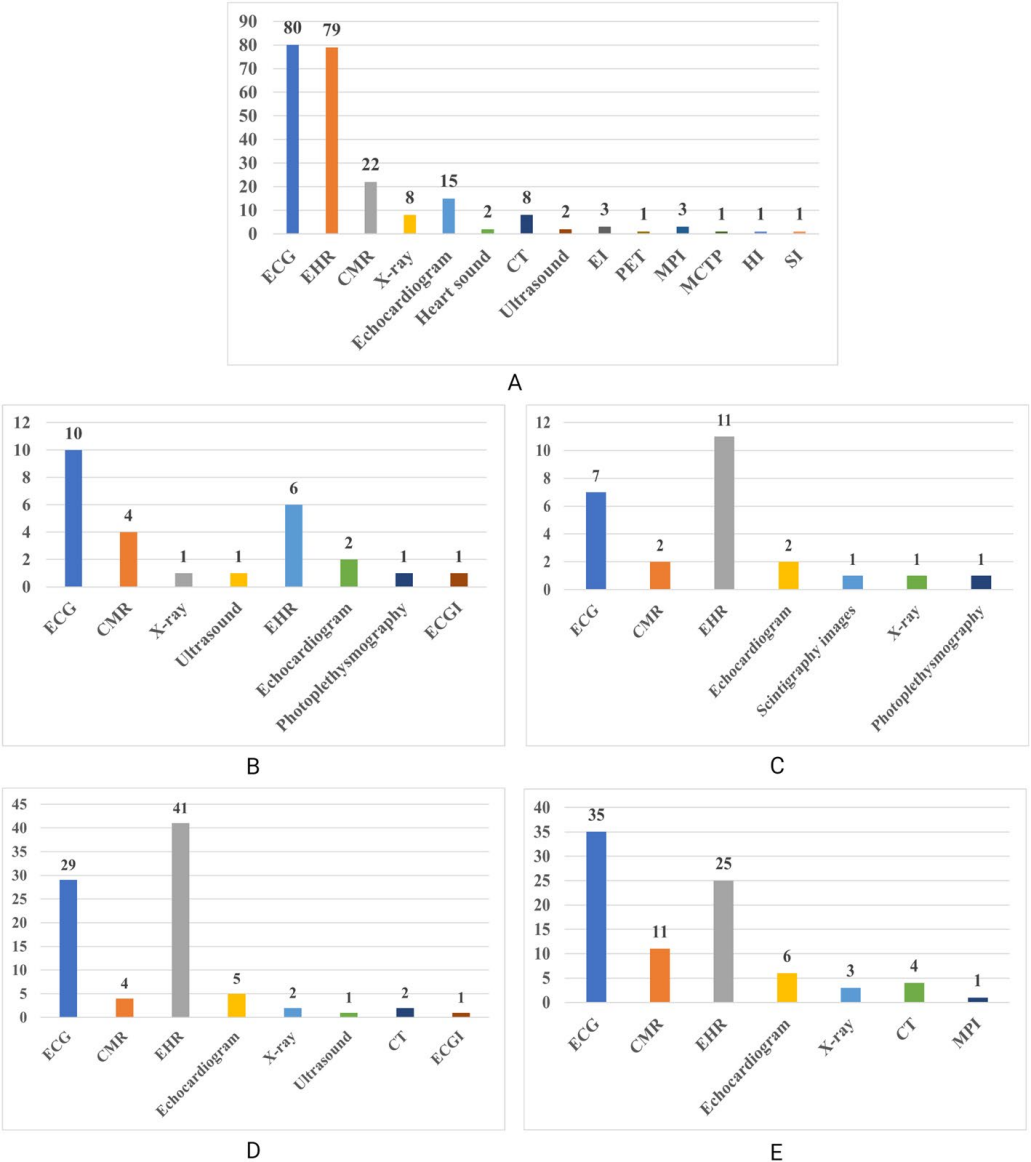
Data modalities used in cardiac studies. **A** All cardiac studies, **B** cardiac studies applied proxy-grounded evaluation approaches, **C** cardiac studies applied expert-grounded evaluation approach, **D** cardiac studies applied literature-grounded evaluation approach, **E** cardiac studies did not apply any kind of evaluation to XAI outcomes. *ECG* electrocardiography, *EHR* electronic health records, *CMR* cardiac magnetic resonance imaging, *CT* computed tomography, *EI* electrocardiographic imaging, *PET* positron emission tomography, *MPI* myocardial perfusion imaging, *MCTP* myocardial computed tomography perfusion, *HI* histology images, *SI* scintigraphy images

Fig. 3 provides an overview of the data modalities used in the studies included in the review and for each category of evaluation approaches. ECG and EHR were the most frequently used data, followed by CMR and echocardiogram. EHR data includes (in our review) cognitive tests, lab tests metrics and any data not considered in imaging, ECG or sound data. ECG and EHR-related patient health information acquired through questionnaires are more readily accessible compared to imaging data, and particularly, CMR, which can be expensive and time consuming. Nonetheless, CMR remains the gold standard for assessing the cardiac structure and function due to its ability to provide unique, in-depth information not attainable by other means. The availability of large biomedical repositories, such as the UK Biobank (Petersen et al. 2015), might result in an increase in the number of studies using CMR data in the coming years. It is worth mentioning that many studies (13) employed multiple data modalities, such as ECG and EHR, ECG and CMR, simultaneously. This explains why the total count of papers for each data type exceeds the total number of studies included in the review.

**Fig. 4:**
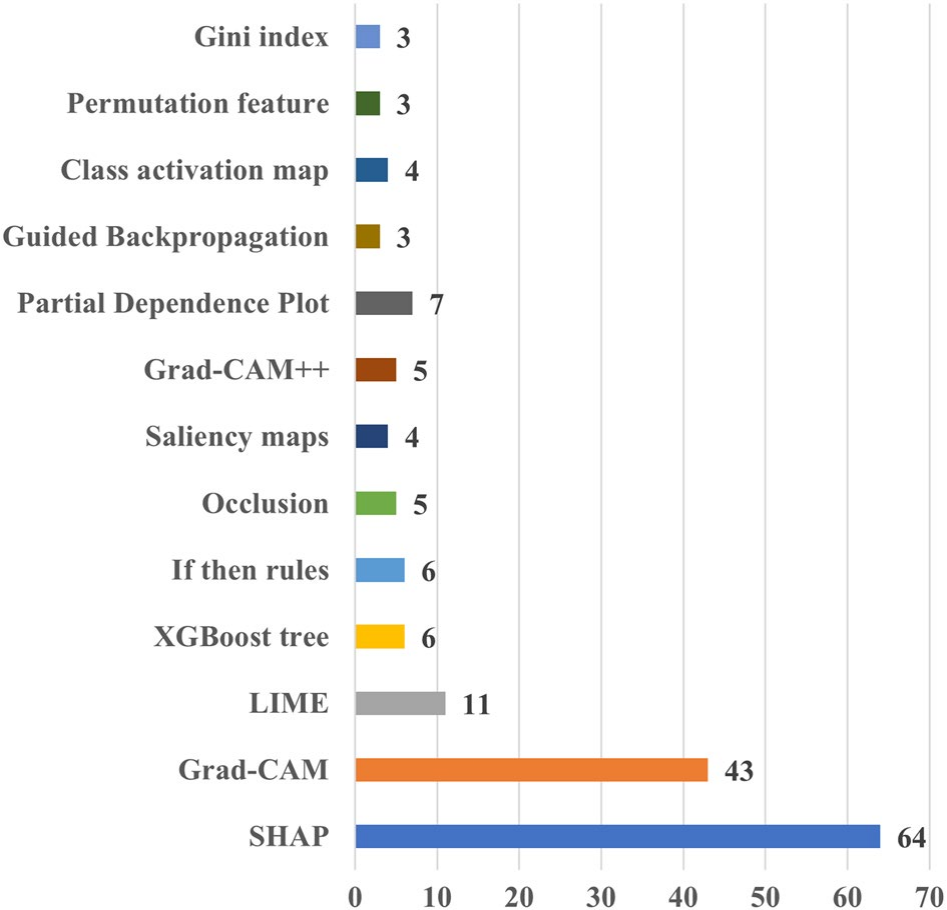
Distribution of the number of cardiac studies employing different XAI methods. *Grad-CAM* Gradient-weighted Class Activation Mapping, *LIME* Local Interpretable Model-agnostic Explanations, *SHAP* Shapley Additive Explanations

Figure 4 provides the number of papers according to XAI method employed. It shows that the majority of the papers applied SHAP, followed by Grad-CAM and LIME. This can be attributed to the versatility of SHAP and LIME, which can be applied to both imaging and tabular data, as those found in EHR data. On the contrary, Grad-CAM can be applied to imaging and signal (e.g., ECG) data. This is somehow expected because these methods have attracted significant attention across various domains, including cardiac research. In addition, their ease of implementation, facilitated by publicly available packages and in multiple programming languages, has further contributed to their popularity. It should be noted that the figure shows the most frequently used XAI methods in cardiac studies, rather than an exhaustive list. In addition, there exist many studies that applied more than one XAI method in their analysis. For more details on the used XAI methods in cardiac studies, please refer to Table S2.

**Fig. 5:**
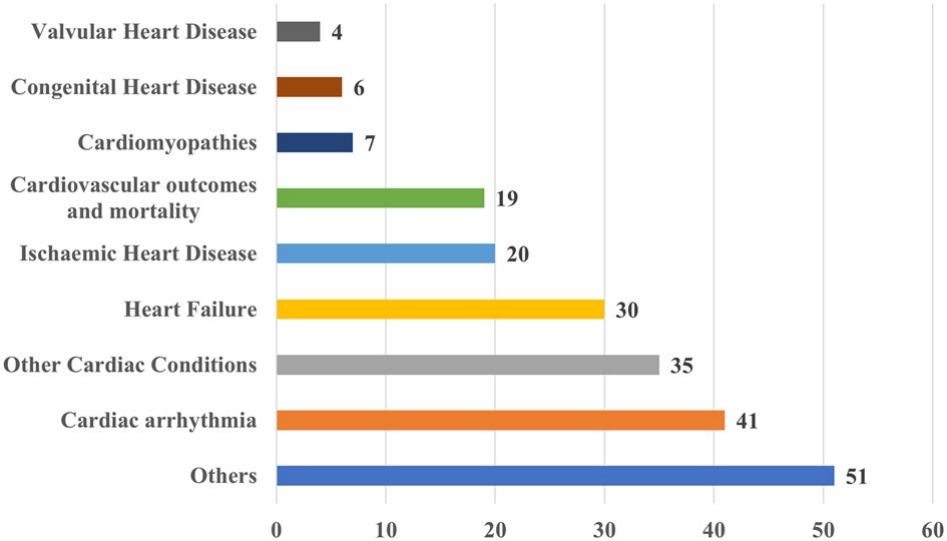
The distribution of the diseases targeted in cardiac studies

Figure 5 shows the distribution of studies depending on their primary area of focus. Articles predominantly concentrating on specific diseases were organized according to their principal disease domains. Specifically, the studies included in the “Cardiac Arrhythmia” group explored various forms of bradyarrhythmia and tachyarrhythmia, as well as related-treatments such as ablation. The “Cardiomyopathies” group encompassed studies focusing on non-ischemic cardiomyopathies. The “Heart Failure”, “Valvular Heart Disease”, and “Congenital Heart Disease” groups comprised works specifically centered around those respective conditions. Additionally, the “Other Cardiac Conditions” category covered a wide range of topics, including stroke, peripheral artery disease, pregnancy, pulmonary hypertension, and other cardiac conditions. Some articles, rather than focusing on specific disease domains, primarily addressed tasks such as image segmentation, detection of cardiac abnormalities, and imaging or ECG-based phenotyping. These articles were collectively categorized under the label “Others”.

The figure shows that cardiac arrhythmia (41 studies) stands out as the most frequently studied cardiac condition. This is probably due to the fact that cardiac arrhythmia can be effectively studied by means of ECG data, which is readily obtainable, and the most common data modality used in the reviewed studies. Heart failure is the second most examined condition, encompassing 30 works. This is primarily attributed to the feasibility of investigating heart failure using non-imaging EHR data which ranks as the second most prevalent data type used in the reviewed studies.

For the remainder of this review, we will group the papers based on the category of evaluation approach applied to their XAI outcomes. In total, we have identified four distinct evaluation approach categories for the cardiac domain: (i) expert-grounded, (ii) proxy-grounded, (iii) literature-grounded, and (iv) none. Papers that relied on cardiologists or clinicians to assess the outcome of XAI were classified as part of the expert-grounded category. Studies using any proxy, statistical method, or other quantitative metrics to evaluate the XAI outcome fell into the proxy-grounded evaluation category. Literature-grounded evaluation included the works where findings from previous publications were used to assess the outcome of XAI. Typically, these works cite previous publications to support their findings. The last group included those works that did not apply any kind of evaluation to the XAI outcome.

**Fig. 6:**
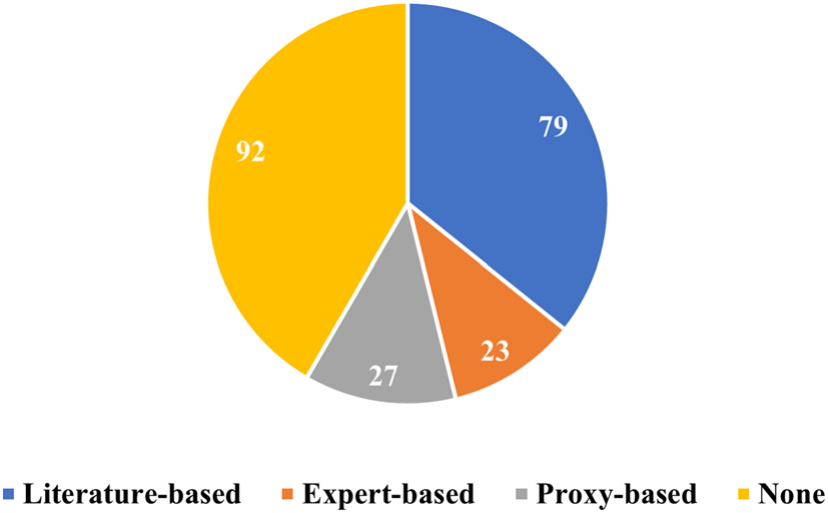
Distribution of the number of papers across four categories of XAI evaluation approaches: (i) literature-grounded, (ii) expert-grounded, (iii) proxy-grounded, (iv) none

Figure 6 shows the distribution of papers employing different evaluation methods to XAI outcomes. The figure highlights that most papers did not apply any evaluation method, followed by those that applied literature-grounded evaluation. In addition, it shows that expert-grounded methods were less frequently employed than other methods. Notably, 8 studies used two different evaluation methods simultaneously, and they are represented in both categories within the figure.

In addition, we have also assessed whether the findings derived from the XAI outcome were in line with the results of the evaluation method. For instance, if an XAI model identified a specific region in CMR as the most informative region for distinguishing between control and heart failure, and this aligned with the expert’s opinion or the applied proxy, it was considered as a match between the XAI outcome and the evaluation approach outcome. Similarly, a mismatch would be recorded if the outcome of the XAI and the evaluation approach did not concur. Cases, where only part of the explanation aligned with expected or established knowledge, are labeled as partial matches.

**Fig. 7:**
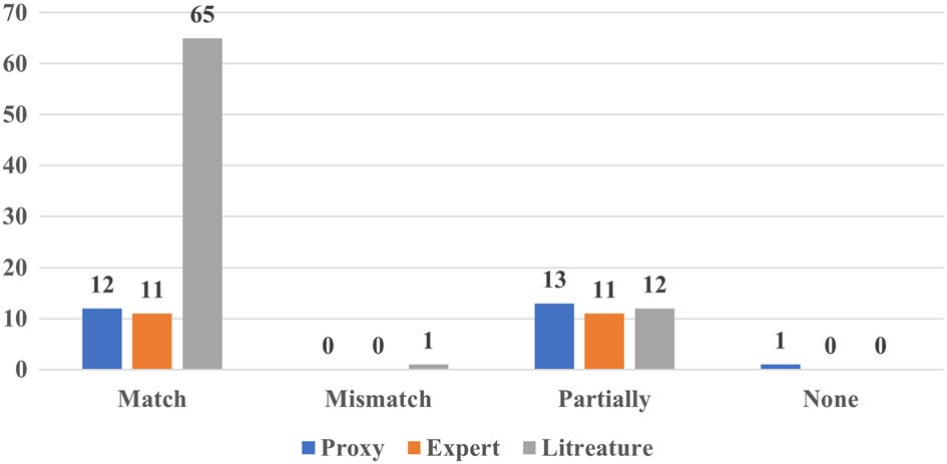
Matching the outcome of the evaluation with the outcome of XAI

In this context, Fig. 7 illustrates that the results most evaluation approaches aligned with the outcomes of XAI. This alignment is particularly evident in the literature-grounded approach as this is the most used evaluation approach. Remarkably, only one study (Aufiero et al. 2022) deviated from this pattern, as its XAI outcomes contradicted prior findings.

## A review of XAI evaluations in cardiology

The following four sections discuss the papers that applied an evaluation method to assess the effectiveness of the used XAI algorithm. Moreover, we provide statistics and tables with information regarding the utilised data types and XAI methods, grouped by the evaluation approach employed.

### Expert-grounded evaluation in cardiac applications

Twenty-three papers relied on expert-grounded evaluations to assess the outcomes of their XAI methods, either alone or in combination with proxies and literature-grounded approaches (Table 2). The experts were represented by cardiologists, physicians or clinicians with different years of experience.

Differently from proxy-grounded evaluations, a greater variety of XAI methods could be found in these 23 reviewed papers, including “if-then” rule, SHAP, Grad-CAM and Saliency maps. The outcomes of the XAI were mostly inline with what was expected by the experts. In particular, the outcomes of eleven works were fully inline with what was expected, while the remaining twelve were partially inline.

**Table 2:** Summary of studies relying on expert-grounded evaluation approaches (used data and XAI methods)

Ref.	XAI method	Data	Specialist	No	Y
Zhang et al. (2019b)	Attention vectors	EHR	Cardiologists	–	–
Vazquez et al. (2021)	SHAP	EHR	Cardiologists	2	
Zhang et al. (2021)	Guided saliency, DeepSHAP, Integrated gradients	PhPM	Experts	–	–
Sangroya et al. (2022)	Domain concepts, SHAP	ECG	Cardiologists	–	–
Pičulin et al. (2022)	SHAP	EHR	Medical experts	13	~ 16
Nurmaini et al. (2022)	Grad-CAM, Guided Backpropagation Grad-CAM	Echocardiogram	Fetal cardiologists	3	–
Decoodt et al. (2023)	Grad-CAM++	X-ray	Cardiologists	1	40
Kukar et al. (2011)	If then rules	EHR, ECG	Physicians	4	–
Li et al. (2020)	If then rules	EHR	Medical expert	1	–
Kwon et al. (2018)	Attention mechanism, If then rules	EHR	Experts	2	–
Jin et al. (2021)	Dual-level attention mechanisms	ECG	Clinicians	–	–
Zhang et al. (2019a)	Attention weights	EHR	Cardiologists	–	–
Bahrami et al. (2019)	Saliency maps	CMR	Radiologists	–	–
Pérez-Pelegrí et al. (2021)	Class activation map	CMR	Cardiologists	2	>10
Halme et al. (2022)	Maximum activation maps	Scintigraphy images	Physicians	5	–
Attia et al. (2021)	Correlation	ECG	Physicians	–	–
Wong et al. (2022)	Parsimony plot	EHR	Experts	–	–
Sager et al. (2021)	Expert-enhanced	EHR, ECG	Cardiac electrophysiology	1	–
Hur et al. (2020)	Attention heatmap	EHR	Cardiologists, Internists	3	–
Jones et al. (2020)	Saliency maps	ECG	Cardiologists	1	–
Meng and Xing (2022)	Guideline	EHR	Experts	–	–
Duffy et al. (2021)	Depth map	Echocardiogram	Physicians	–	–
Yoo et al. (2021)	Class attention map	ECG	Cardiologists	–	–

Zhang et al. (2019b), Vazquez et al. (2021) and Zhang et al. (2021) were included as well because they used both proxy- and expert-grounded methods

*No* number of experts, *Y* years of experience, *PhPM* photoplethysmography

In Pičulin et al. (2022), Zhang et al. (2021), Sangroya et al. (2022) and Vazquez et al. (2021), SHAP was used as XAI method alongside with others such as integrated gradients (Zhang et al. 2021) and domain concepts (Sangroya et al. 2022). “If-then” rule was used as XAI method to explain the classification models applied to detect heart failure (Li et al. 2020; Kukar et al. 2011; Kwon et al. 2018).

Table 2 shows that the majority of the studies applied expert-grounded evaluation did not report the number of experts involved in the evaluation, their medical specialty nor the years of experience of the experts. Four studies (Decoodt et al. 2023; Sager et al. 2021; Jones et al. 2020; Li et al. 2020) included one expert in the evaluation without reporting the years of experience apart from one (Decoodt et al. 2023). One study (Pičulin et al. 2022) involved a decent number of experts and experience. They proposed a model to predict the clinical statues 10-years ahead for those experienced hypertrophic cardiomyopathy. They applied SHAP to explain the model and its outcome was evaluated by 13 medical experts with 16 years (SD 8) of experience. Manual segmentation of two cardiologists with more than 10 years of experiences were used to assess the outcome of a class activation map applied to a deep learning model to estimate left ventricle volume (Pérez-Pelegrí et al. 2021). The “If-then” rule was implemented as XAI method in Kukar et al. (2011) for a model diagnosing patients with coronary artery disease automatically. The proposed method evaluates myocardial scintigraphy imaging and extracts parameters to then be combined in another model for classification matter. To assess the XAI method, four expert physicians assessed the cardiac images and provided the level of coronary artery congestion by attributing values to the different myocardial regions. The model yielded attributes that closely mirrored the assessments offered by the expert physicians and the “If-then” rule.

More details on each study using expert-grounded evaluations are presented in Table S2.

### Proxy-grounded evaluation in cardiac applications

Twenty-seven papers applied proxy-grounded methods, either alone or in conjunction with other approaches, to evaluate the outcomes of XAI methods. The evaluation results of the majority aligned with the outcome of the XAI methods, either fully or partially. No contradiction between the evaluation outcome and the XAI outcome. One paper (Prifti et al. 2021) did not comment or compare the results of the evaluation method and the XAI outcome. Table 3 summarizes the papers that applied proxy-grounded evaluation methods to assess the XAI outcomes. It indicates that ECG and EHR were the predominant data types used, while Grad-CAM and SHAP were the most frequently used XAI methods.

**Table 3:** Summary of the used data and XAI methods in studies employing a proxy-grounded evaluation approach

Ref.	XAI method	Data	Proxy	How does it work
Zhang et al. (2019b)	Attention vectors	ECG	Contribution rate	Calculate the contribution rate of each feature to the model output using simulated and real-world data
Vazquez et al. (2021)	SHAP	EHR	Multivariable Cox regression	Using the informative predictors identified by an XAI to estimate mortality using a survival analysis model
Zhang et al. (2021)	Guided saliency, DeepSHAP Integrated gradients	Photoplethysmography	Congruence, Annotation classification Accuracy, NPV, specificity	The agreement between XAI annotations and expert annotations Perform correlation between model performance metrics and pre-defined explanation metrics
Prifti et al. (2021)	Occlusion	ECG	Occlusion analysis	Similar to ROAR but instead of removing the feature/regions, they were occluded
Pham et al. (2023)	Feature-view attention weights	EHR	Selectivity or RemOve And Retrain (ROAR)	The model should experience sharp reduction if top features identified by an XAI method were removed from the model
Kwon et al. (2021)	Grad-CAM	ECG	Akaike information criterion and the mean decreased Gini	Akaike information criterion is used to assess how a model fit well to data. Gini index is used with random forest model
Abdullah et al. (2023)	LIME	ECG	Local fidelity	How well the XAI method approximated the model locally for a single instance and can be measured through accuracy and F1 score
Karoui et al. (2021)	DirectMap	ECG	Absolute activation time error	The absolute difference between the actual and the calculated activation times
Wall et al. (2022)	SHAP	ECG	Permutation importance	The model should experience sharp reduction if top features identified by an XAI method were shuffled
Wang et al. (2022a)	Grad-CAM	Echocardiogram	Compare segmentation vs heatmap	Compare visually the heat maps against the segmentation target to assess if the model was correctly identified regions significant in the model
Tsuji et al. (2023)	Grad-CAM	X-ray	Attention index	The method uses attention branch network to assess how the generated maps are comparable
Dakshit et al. (2022)	Dynamic Time Warping, Mean Squared Error, SLACK	ECG	Selectivity or RemOve And Retrain (ROAR)	The model should experience sharp reduction if top features identified by an XAI method were removed from the model
Bacoyannis et al. (2021)	Activation map	Electrocardiographic	Difference between the ground truth and the activation maps	Generate n different activation maps and compare the mean and SD between the ground truth activation and the generated activation maps
Kofler et al. (2023)	Dictionary learning	CMR	Stability and generalization	Calculating point wise error and how the model would behave when training and testing on different dataset
Leur et al. (2021)	Grad-CAM++	ECG	Logistic regression	Translate the identified regions by XAI in ECG into quantitative features and then add them to a baseline model using logistic regression, then check if the model perform better
Mokhtari et al. (2022)	Learned weights	Echocardiogram	Average frame distance	Average frame distance calculates the difference between the true and the approximated indices
Tang et al. (2022)	Deep Taylor decomposition	EHR	Mean relevance	Calculate mean relevance between each clinical parameter (predictor) identified by an XAI method and the outcome
Ganeshkumar et al. (2021)	Grad-CAM	ECG	Correlation	Perform correlation between activation maps generated by an XAI method and the variations in the characteristic of the used data (here ECG)
Ogbomo-Harmitt et al. (2023)	Grad-CAM, Occlusions and LIME	CMR	Wilcoxon signed-rank test	Assess the outcomes of several XAI methods by ranking them based on the percentage of identifying informative regions
Panicacci et al. (2019)	Gini index	EHR	Permutation importance, Gini decrease, trees and roots	Gini decrease, trees and roots are related to random forest model
Clough et al. (2019)	Testing with concept activation vectors	CMR	Interpolations	Observe the change in an image domain when pre-defined concepts by an XAI method were interpolated
Beetz et al. (2022)	VAE’s latent space	CMR	Latent space analysis	Calculate the activity of latent space in variational mesh autoencoder
Singh and Sharma (2022)	SHAP, LIME, Grad-CAM	ECG	Dice loss, linear unit and gaussian filter	Calculate a similarity coefficient between the outcome of several XAI methods
Le et al. (2023)	Grad-CAM, SHAP	ECG	Sanity check	A proxy to assess whether the explanation related to the model or to the data
Karri et al. (2021)	SHAP	EHR	Logistic regression coefficients	Train a model with an XAI and a logistic regression model. Then, compare the informative predictors from the XAI method with the coefficient value
Sakai et al. (2022)	Graph chart diagram	Ultrasound	Abnormality score	Compare the area of visualized region in normal and abnormal cases
Huynh et al. (2022)	Saliency maps, Grad-CAM and XRAI	X-ray	Lung area attention and lung blur sensitivity	The proportion of overlapping between the XAI maps and the masks of segmentation generated by the U-net model Ronneberger et al. (2015)

More precisely, out of the 27, six works (Wall et al. 2022; Zhang et al. 2021; Singh and Sharma 2022; Le et al. 2023; Karri et al. 2021; Vazquez et al. 2021) used SHAP as the XAI method across different domains, including stroke, arrhythmia, atrial fibrillation and hospital mortality. The results of the XAI evaluation were either fully or partially inline with the outcome of SHAP. The evaluation metrics included permutation importance, accuracy reduction, sanity check and checking the value of the logistic regression coefficients to assess whether a feature is informative or not.

Selectivity or RemOve And Retrain (ROAR) method was applied to two works (Pham et al. 2023; Dakshit et al. 2022) to evaluate if the model identified the correct features that drive model outcome. Another two works (Wall et al. 2022; Prifti et al. 2021) followed the same criterion but instead of removing the top features, they were permuted. Statistical methods and models were used to evaluate the outcome of XAI methods. Permutation importance served as a proxy for evaluating the list of informative predictors produced by SHAP to estimate cardiac age using ECG features (Wall et al. 2022). The proxy results confirmed that the identified features by SHAP have a significant impact on the model outcome. Another assessment of SHAP involved using the coefficient values of logistic regression as a proxy (Karri et al. 2021). In this study, multiple models were developed to classify patients with postoperative atrial fibrillation. For the best performing model, SHAP was applied to obtain the most important features in the model’s decision. Moreover, the authors compared the list of the features provided by SHAP with the coefficient values produced by logistic regression. They found that there is a partial match between the coefficient value of the features and their index of order in the list provided by SHAP.

Grad-CAM was employed to explain a multilabel classification model distinguishing between healthy control and eight cardiac diseases using ECG (Ganeshkumar et al. 2021). To assess whether the model learnt relevant features, they calculated the correlation between the activation map provided by Grad-CAM for each disease and their respective variations in the ECG. The results of the correlation confirmed that the model decision was indeed driven by the right features in ECG. In another study, SHAP, LIME and Grad-CAM were used to explain a model distinguishing between individuals with arrhythmia from control using ECG (Singh and Sharma 2022). To evaluate the outcome of the XAI methods, they used rectified linear unit and gaussian filter to smooth the generated feature maps from each XAI method. Subsequently, they segmented the ECG into windows and fed it to each XAI method to generate saliency plots for each class. Finally, heatmaps were generated based on the values of the feature importance. This approach confirmed whether the model effectively searched in the ECG segments during classification. More details on each study that used proxy-grounded approach is presented in table TableS2.

### Literature-grounded evaluation in cardiac applications

**Fig. 8:**
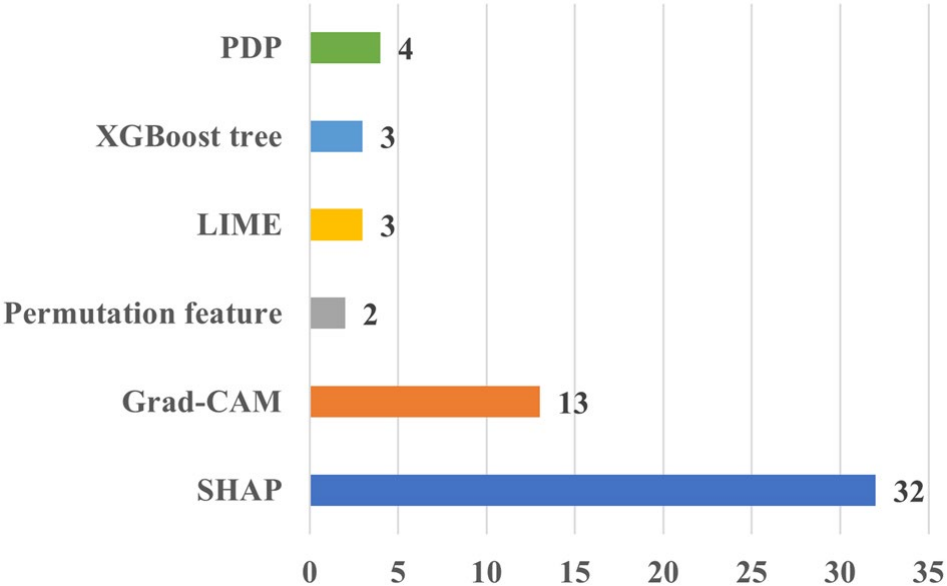
The number of the XAI methods used in cardiac applications. *Grad-CAM* Gradient-weighted Class Activation Mapping, *LIME* Local Interpretable Model-agnostic Explanations, *SHAP* Shapley Additive Explanations

Most of the papers (seventy-nine) included in this review used a literature-grounded approach to evaluate the performance of XAI(Table 4). SHAP and Grad-CAM were the most common XAI methods applied in these studies providing literature-grounded evaluations (Fig. 8).

**Table 4:** Summary of the used data and XAI methods for those applied literature-grounded evaluation approach

XAI method	Data	References
SHAP	ECG	Agrawal et al. (2022), Xiao et al. (2023), Zhang et al. (2021), González et al. (2022), Oliveira et al. (2023), Portella et al. (2022), Ding et al. (2023), Gkontra et al. (2023)
	EHR	Duval et al. (2023), Wang et al. (2021b, 2022d), Forte et al. (2022), Kor et al. (2022), Shi et al. (2022), Zeng et al. (2021), Shah et al. (2022), Peng et al. (2022), Haque et al. (2022), Hamatani et al. (2022), Kucukseymen et al. (2022), Shin et al. (2021), Li et al. (2022), Sun et al. (2022), Hong et al. (2022), Wu et al. (2023), Vazquez et al. (2021), Niu et al. (2023), Lu et al. (2021), Killian (2023), Ding et al. (2023), Stabellini et al. (2023), Miranda et al. (2023), Zeng et al. (2024)
	CMR	Kucukseymen et al. (2022)
	Others	Hamatani et al. (2022), Lo Iacono et al. (2023)
Grad-CAM	ECG	Aufiero et al. (2022), Melzi et al. (2021), Cho et al. (2021), Kwon et al. (2021, 2020), Zeng et al. (2022), Alkhodari et al. (2022), Ho and Ding(2022), Lopes et al. (2021), Vijayarangan et al. (2020)
	Others	Ragnarsdottir et al. (2022)
Grad-CAM++	ECG	Jiang et al. (2022), Markov et al. (2023)
LIME	EHR	Haque et al. (2022), Sun et al. (2022), Hong et al. (2022)
PDP	EHR	Sun et al. (2022), Gandin et al. (2023), Lisboa et al.(2022), Patel et al. (2021), Rauf et al. (2023)
GBM	ECG	Ding et al. (2023)
	EHR	Shin et al. (2021), Wu et al. (2023), Ding et al. (2023), Rauf et al. (2023)
Other	Shi et al. (2022), Patel et al. (2021), Pham et al. (2023), Michel et al. (2021), Rao et al. (2022), Miran et al. (2021), Loncaric et al. (2021), Shi et al. (2018), Ghorbani et al. (2020), Wong et al. (2022), Khurshid et al. (2022), Wouters et al. (2023), Kawakami et al. (2022), Sang et al. (2022), Lindow et al. (2022), Wongvibulsin et al. (2020), Chen et al. (2020), Sammani et al. (2022), Wu et al. (2023), Alabed et al. (2022), Tamarappoo et al. (2021), Wang (2021a), Schrutka et al. (2022), Hong et al. (2019), Bodini et al. (2021), Doborjeh et al. (2022), Chen et al. (2023a), Saito et al. (2022), Tong et al. (2019), Qu et al. (2022), Chen et al. (2023b)	

Kwon et al. (2021) and Pham et al. (2023) were included again because they applied proxy and literature-grounded evaluation, Wong et al. (2022) applied expert and literature-grounded approaches while Vazquez et al. (2021) implemented the three approaches

Going into more details of some of these studies, Aufiero et al. (2022) identified new ECG features using a DL model combined with Grad-CAM in congenital long QT syndrome patients. Their approach identified the QRS complex as the most relevant feature that dominated the classifier decision, a novel finding that had never previously been reported in this condition. Another study (Gandin et al. 2023) used EHR to devise a deep learning model for predicting the risk of developing heart failure in diabetic patients. To understand the model outcome and the role of the included features, the authors adopted (Gandin et al. 2023) partial dependence plot (PDP) (Greenwell et al. 2018), which identified as highly relevant features such as diuretics, diabetes duration, arterial hypertension and Charlson comorbidity index. As acknowledged by the authors themselves, these features are well-known and have been previously reported in heart failure patients.

An ML model was developed to distinguish individuals with heart amyloidosis from hypertrophic cardiomyopathy using EHR and echocardiography data (Wu et al. 2023). They implemented information gain of XGBoost to identify the most important features in the model. Previous findings support significant predictors to disseminate between the two conditions. More details of each study used literature-grounded approach is represented in Table S2.

### No evaluation method

Ninety-two papers included XAI in their framework but did not apply any kind of evaluation to assess the XAI performance and corresponding outcomes. Table 5 summarizes the used data and the XAI methods. ECG data were the most common ones, followed by EHR and CMR (Fig. 3).

**Table 5:** Summary of the used data and XAI methods for those did not apply any kind of evaluation to the outcome of XAI methods

XAI method	Data	References
SHAP	ECG	Rouhi et al. (2021), Jekova et al. (2022), Rashed-Al-Mahfuz et al. (2021), Angelaki et al. (2021), Anand et al. (2022), Wickramasinghe and Athif (2022), Soto et al. (2022), Ukil et al. (2023)
	EHR	Kogan et al. (2023), Smole et al. (2021), Moreno-Sanchez (2020), Fan et al. (2022), Vaulet et al. (2022), Abraham et al. (2022), Goswami et al. (2023), Pieszko et al. (2023), Guleria et al. (2022), Dong et al. (2023), Mahajan et al. (2023), Zhou et al. (2023)
	CMR	Salih et al. (2023a)
	Others	Soto et al. (2022), Pieszko et al. (2023), Singh et al. (2023), Lagopoulos and Hristu-Varsakelis (2022), Lee et al. (2023)
Grad-CAM	ECG	Wang et al. (2022b), Vafaeezadeh et al. (2022), Maiorana et al. (2023), Sawano et al. (2022), Cao et al. (2022), Sharma et al. (2022), Apama et al. (2022), Lee and Shin (2021), Yue and Zhu (2023), Cetin et al. (2023), Sangha et al. (2022)
	EHR	Cetin et al. (2023)
	CMR	Cetin et al. (2023)
	Others	Singh et al. (2023), Choi et al. (2023), Sanjeevi et al. (2023), Maiorana et al. (2023), Singh et al. (2022), Jiao et al. (2022), Ovalle-Magallanes et al. (2020)
Grad-CAM++	ECG	Fang et al. (2022)
	Others	Makimoto et al. (2022)
LIME	ECG	Rouhi et al. (2021), Abdullah et al. (2022), Sbrollini et al. (2022), Chen et al. (2023)
	Others	Ly et al. (2022)
PDP	EHR	Goswami et al. (2023)
	Others	Lee et al. (2023)
If then rule	EHR	Sannino et al. (2021), Roseiro et al. (2023)
	Others	Soares et al. (2020)
GBM	ECG	Ye et al. (2021)
	EHR	Commandeur et al. (2020)
	Others,	Commandeur et al. (2020)
Other	Rouhi et al. (2021), Jekova et al. (2022), Moreno-Sanchez (2020), Goswami et al. (2023), Sun et al. (2020), Bahani et al. (2021), Rueda et al. (2022), Liu et al. (2023), Wesołowski et al. (2022), Liu et al. (2023), Wang et al. (2022c), Nankani and Baruah (2022), Zhao et al. (2023), Wang et al. (2021c), Diaz Ochoa et al. (2023), Fang et al. (2020), Zhang et al. (2023), Xing et al. (2021), Kan et al. (2023), Yin et al. (2019), Painchaud et al. (2022), Bhardwaj et al. (2023), Lin et al. (2020), Gee et al. (2019), Mousavi et al. (2020), Liang and Guo (2023), Johnson et al. (2022), Guo et al. (2020), Ma et al. (2019), Janik et al. (2021), Biffi et al. (2018), Hu et al. (2020), Beetz et al. (2022), Nguyen et al. (2022), Nankani and Baruah (2021), Wu et al. (2022), Yang et al. (2022), Hu et al. (2023), Gao et al. (2023), Huang et al. (2023), Valvano et al. (2022), Brisimi et al. (2018)	

**Fig. 9:**
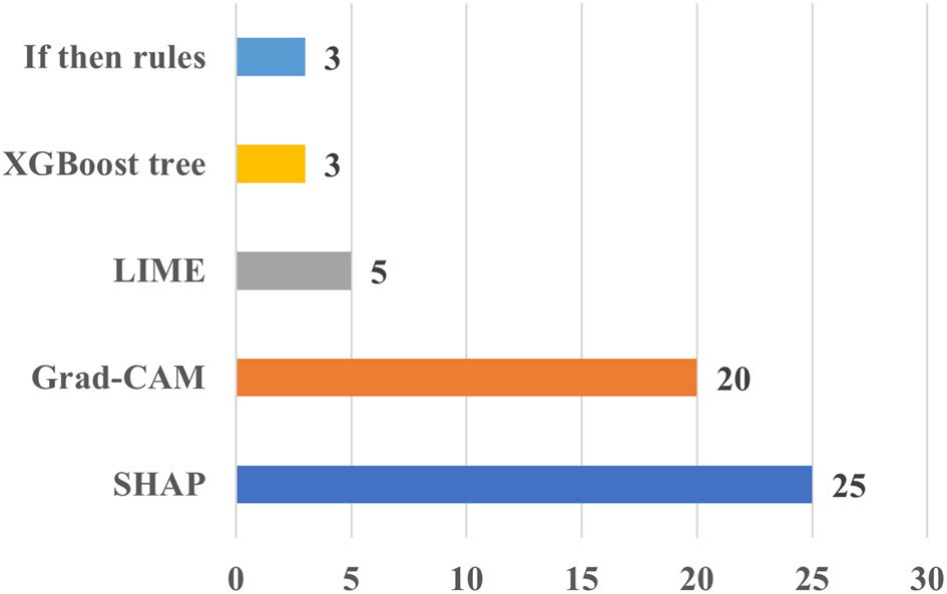
The number of the XAI methods used in cardiac applications. *Grad-CAM* Gradient-weighted Class Activation Mapping, *LIME* Local Interpretable Model-agnostic Explanations, *SHAP* Shapley Additive Explanations

In terms of XAI methods, SHAP (25) and Grad-CAM (20) were the prevalent XAI choices for these studies, similarly to what found in the other papers employing XAI in combination with some kinds of evaluation (Fig. 9).

## Discussion

In this section we detail key observations from our review of XAI research in cardiac study algorithms. We list challenges that XAI developers or users might face and we provide recommendations for the development of XAI, where possible.

### Notes on the cardiac studies

A range of data modalities, model architectures, cardiac conditions, XAI and evaluation approaches were present in the studies included in this review and are summarised below.

1. **Data modalities:** Most studies in this review used either ECG or EHR data. ECG data may be acquired rapidly, easily, and cheaply compared to imaging data such are CMR. However, ECGs report the electrical function of the cardiac, while CMR imaging provides structural and functional information. The modality/modalities for the data leading to an optimal model result (as determined by accuracy for example) will vary on a case-by-case basis depending on the modelling objective.
2. **Model architectures:** Most of the algorithms that were used were binary classification models. A small number of studies used regression models to estimate a continues variable. We note that regression models can also be used to discriminate between two conditions through (1) comparing against a normal reference range for a specific phenotype (e.g. left ventricular ejection fraction, left ventricular end-systolic volume), or (2) predicting a continues variable (e.g cardiac age, left ventricular mass) for a cohort free of cardiac diseases with validation on a cohort with the cardiac condition under examination.
3. **Cardiac conditions:** Arrhythmia and heart failure were the most examined conditions, which may be driven the availability of ECG data. Although coronary (ischemic) heart disease is the most common cardiac disease worldwide (British Heart Foundation 2023), it was [the least] investigated compared to other cardiac conditions. This is because we used broader terms such as “cardiac” and “heart” to encompass a wide array of studies within the field, rather than focusing narrowly on specific conditions like coronary artery disease (CAD). This approach might have probably limited the number of papers specifically focused on CAD. Incorporating more specific keywords could have increased the CAD-related publications, but that would also necessitate including a variety of terms for other cardiac conditions, which was beyond the scope of our paper. Most of the studies investigated heart failure used EHR as the input data, as mentioned above. Other cardiac conditions may be less investigated due to reduced incidence and/or reduced availability of the specific data modalities necessary.
4. **XAI model:** Most of the XAI reviewed here used the SHAP and Grad-CAM methods followed by LIME. These methods have contributed significantly to the body knowledge of XAI, but they are imperfect and have their own drawbacks including concerning against adversarial attacks and localize multiple occurrences within an image (Slack et al. 2020; Chattopadhay et al. 2018). The results produced by these three methods are easy to understand and interpret, which may have enhanced the uptake of the methods, as could the ready availability of software code and packages.
5. **Evaluation approach:** 43% of the papers did not use any kind of evaluation approach to assess the performance of XAI. In addition, 37% used literature-grounded approach followed by 11% using proxy-grounded approach and 11% using expert-grounded evaluation. The literature-grounded approach was the most used one due to the ease of carrying out reviews using different repositories including IEEE Xplore, Web of Science and PubMed. The expert-grounded approach is the least used because it specialist reviewer time is costly and time-consuming to carry out on all XAI outputs. The proxy-grounded approach is still under development which may explain why only 11% papers evaluated XAI performance using this approach. The majority of studies did not evaluate the model results which may happen when developing a new XAI model or examining a rare condition where literature and expert-grounded approaches might not be available.
6. **Expert-grounded evaluation:** The authors of the papers using the expert-grounded approach to assess the XAI outcomes included physicians (Halme et al. 2022), clinicians (Jin et al. 2021) and internists (Hur et al. 2020), categories of professionals likely experienced in the relevant cardiac diseases. Notably, however, only three papers (Pérez-Pelegrí et al. 2021; Pičulin et al. 2022; Decoodt et al. 2023) out of the 23 in total mentioned the number of years of relevant experience when evaluating XAI performance. One study used 13 experts to assess XAI (Pičulin et al. 2022) and four used one expert (Decoodt et al. 2023; Sager et al. 2021; Jones et al. 2020; Li et al. 2020), while the majority did not specify the number of experts employed.
7. **XAI evaluation outcome:** Enormous number of the papers that applied any kind of evaluation approach got a match between the XAI outcome and the outcome of the used evaluation approach, especially with literature-grounded approach as it is the most used one. The reason behind that could be the examined cardiac conditions are very complex (e.g. heart failure) and there is more than one factor affecting the condition significantly and simultaneously. Accordingly, even if the outcomes of two XAI methods vary for the same condition, yet they still carry informative predictors for that condition and match with the previous findings or with expert opinions.

### Model performance vs model explainability

Ideally, model performance and explainability would be defining features of a good model. Here we consider the relationship between these two characteristics.

1. **Inaccurate perception:** A common perception is that the models with high performance are less explainable while more explainable models are those with a lower performance. However, there are many approaches to explainability each with different applicability and utility and this perception requires qualification. The defined aim of the explainability is to produce a framework for the end-user to understand how the results are produced using granular features, as opposed to the complex internal workings of the model architecture. The utility of a given explainability output for a specific end-user is subjective. The results of a recent empirical study (Herm et al. 2023) showed that the trade-off curve between model performance and model interpretability is not gradual.
2. **Explanation form:** Explanations may comprise: lists of informative predictors; highlighted informative regions within an image; uncertainty quantifiers; “what-if” rules; and the probability of an instance belong to a specific class. Some explainability metrics may be more significant than others in a given domain. For instance, uncertainty quantifiers might be more significant than a list of informative predictors in a model using few numbers of predictors. Not all explainability metrics will be suitable for a given model, even if it is of high performance.
3. **Trade-off between model performance and model explainability:** In some cases simple but adequate models with more detailed explanations might be preferable to comparably performing but complex models with reduced level of explanation. One factor in the decision of which model to use might be the domain.
4. **Explanation perceived by end-user:** Model metrics such as accuracy, F1 or mean absolute error are objective qualifiers of a model. However, as XAI methods are means to explain the model for end-users, such explanations are subjective as it is left to each end-user to assess utility.
5. **Simple tasks:** Classification or regression using simple tabular data can be performed using either simple or complex models, with either typically having similar performance and, in some cases the, former outperform the latter (Herm et al. 2023). Accordingly, it is recommended that simple models should be applied in such cases when they are more explainable.

### A reasonable implementation of XAI

It is difficult to determine which of the reviewed papers applied a more reliable and understandable XAI method to end users cardiologists. This is because understanding the outcome of XAI is rather subjective which might differ from a cardiologist to another. Moreover, applying a specific kind of XAI method or evaluation approach is subject to the available data and resources to evaluate the outcomes. However, in Zhang et al. (2021) we believe the authors implemented and evaluated XAI in a robust and reasonable way. First of all, they applied three XAI methods that are Guided saliency, DeepSHAP, and Integrated Gradients. It is recommended that different XAI methods should be implemented to compare and contrast the XAI outcomes from different methods because each method has its own limitations. Secondly, they applied two approaches to evaluate the outcome of XAI that are: expert and proxy-based evaluation. Indeed, it is vital to include the experts in the evaluation of XAI outcome in this stage. On the other hand, including proxy-based approach would assure to evaluate the outcome of XAI objectively. They compared the annotations of the three XAI methods with experts’ annotations using two metrics named Congruence and Annotation Classification. Finally, they performed correlation between the explainability metrics and the model performance including accuracy and specificity to explore whether the used explainability metrics are consistence with the model performance. As XAI still in the development stage and not mature yet, we believe that what the authors did resulted in a more reliable and trusted XAI outcome to the end users.

### Challenges and solutions

The performance of a machine learning model depends on several aspects including sample size, normative features, redundant features, noise, feature collinearity, model architecture, optimisation method, training and validate approaches, and other factors. We list recommendations that might help improving model performance and allow XAI to be evaluated fairly below.

1. **Sample size:** Both simple and complex machine learning models may perform better with larger datasets and variety of data which may be difficult to obtain in the healthcare domain. In addition, unbalanced data happens frequently in healthcare data which might negatively impact the model performance or generalizability. In these cases data augmentation and transfer learning might help to increase sample size, balance the data and train the model on sufficient number of samples.
2. **Use different models:** XAI methods are model-dependent which means their utility depends on the performance of the model being explained. Model performance will depend on the underlying data distribution. In addition, some models might be more or less affected by sample size and the number of features than others. There is not always a standard way to apply a specific model architecture to specific data. This can be examined through exploring variety of models covering simple and complex models: the architecture that achieves a better performance then can be used with XAI to explain how the model works, respecting the premise that, performance being comparable, simpler models are preferable.
3. **Apply several XAI:** XAI methods are not perfect and vary in bias toward specific data, impact of collinearity among predictors, image resolution and lack of causality. Some methods may be more suitable in some domains than others or work with better with specific classes of models. Furthermore, ultimately, it is the end user who determines which XAI method is more meaningful to them.
4. **Evaluation approach and the domain:** It is hard to decide which evaluation approach to choose when evaluating the performance of a given XAI method. This choice may be domain dependent, for example proxy-grounded approaches might be preferable when testing a new product or service where misleading explanations might not be expensive (or harmful). In our opinion, including cardiologists (expert-grounded) alone or alongside with other metrics (proxy-grounded) in cardiac studies to explain the model is still of crucial importance: XAI evaluation is immature and under active development (Salih et al. 2023b).
5. **Blind evaluation:** We believe that including experts in the evaluation of XAI models is valuable. However, the evaluation process should itself be well designed and blinded so that the experts (e.g. cardiologists) provide their explanations and expectations before knowing the outcome of XAI to reduce this source of bias. In addition, in some of the reviewed papers, the number of the physicians whose evaluated XAI was low (Pérez-Pelegrí et al. 2021) which questions the reproducibility of their evaluation. While it might be difficult to include many experienced cardiologists as expert evaluators, if an expert-grounded approach is considered, there should be adequate number of experts with qualified experience to assess XAI to ensure it is reliable and reproducible.
6. **Collinearity:** Many factors including high blood pressure, smoking, alcohol, physical activity, obesity, and diabetes increase the risk of stroke and other cardiac disease. These factors may be related, for example: physical activity and obesity; smoking and alcohol use; and high blood pressure and diabetes. These factors have different clinical interpretations and are often used together in machine learning models when studying cardiac disease. However, XAI methods might be affected by collinearity among predictors and provide unrealistic or biased explanation (Salih et al. 2024). Different attempts and solutions have been proposed to deal with the collinearity including (Salih et al. 2022, 2024; Aas et al. 2021) which should be considered if feature selection or dimensionality reduction method is not employed.
7. **Use literature as confirmation:** Literature-grounded evaluation is a straightforward and immediate way to assess the performance of XAI models. The availability of large biomedical repositories, including the UK Biobank (Petersen et al. 2015) which contains both ECG and CMR data in around 100,000 participants, has increased the volume of cardiac studies published. However, comparing published results requires that the impact of dataset, sample size and model differences are considered as these all affecting the performance of XAI. In addition, if by one side the agreement with previous literature enforces the plausibility of the results, by the other it should not be considered as a must, because this would lead to discard new yet unpublished findings.

## Conclusion

The rapid success in data processing, availability of large biomedical and healthcare datasets and repositories, and variety of XAI models led to an increase the adoption of interpretable models applied to cardiac studies. However, XAI evaluation is not mature yet and still in the development process and might take more time to be adopted in clinical decision-making. In this work we reviewed XAI evaluation approaches applied to cardiac studies. XAI evaluation is an essential step in XAI modelling specially in healthcare sectors. Including a reasonable number of experienced cardiologists to assess the performance of XAI is indispensable even if other approaches of evaluation are adopted. Including experts in the evaluation of XAI provides several key benefits that are: I) making the model more trustful, II) assisting to improve XAI performance and making it more transparent and III) avoiding biased decision derived by the model. Although XAI evaluation is still to be improved and tested on different datasets, machine learning models and XAI methods, their contributions hold high value and push the process toward more mature approaches and metrics.

## Supplementary Information

Below is the link to the electronic supplementary material.

Supplementary file1 (DOCX 14 KB)

Supplementary file3 (XLSX 47 KB)

## Data Availability

No datasets were generated or analysed during the current study.

## References

[CR1] Aas K, Jullum M, Løland A (2021) Explaining individual predictions when features are dependent: more accurate approximations to Shapley values. Artif Intell 298:103502

[CR2] Abdullah TA, Zahid MSBM, Tang TB, Ali W, Nasser M (2022) Explainable deep learning model for cardiac arrhythmia classification. In: 2022 International conference on future trends in smart communities (ICFTSC). IEEE, pp 87–92

[CR3] Abdullah TA, Zahid MSM, Ali W, Hassan SU (2023) B-LIME: an improvement of lime for interpretable deep learning classification of cardiac arrhythmia from ECG signals. Processes 11(2):595

[CR4] Abraham VM, Booth G, Geiger P, Balazs GC, Goldman A (2022) Machine-learning models predict 30-day mortality, cardiovascular complications, and respiratory complications after aseptic revision total joint arthroplasty. Clin Orthop Relat Res 480(11):2137–2145 10.1097/CORR.0000000000002276 PMC9555902 35767804

[CR5] Agrawal A, Chauhan A, Shetty MK, Gupta MD, Gupta A et al (2022) ECG-iCOVIDNet: interpretable ai model to identify changes in the ecg signals of post-covid subjects. Comput Biol Med 146:105540 35533456 10.1016/j.compbiomed.2022.105540 PMC9055384

[CR6] Alabed S, Uthoff J, Zhou S, Garg P, Dwivedi K, Alandejani F, Gosling R, Schobs L, Brook M, Shahin Y et al (2022) Machine learning cardiac-MRI features predict mortality in newly diagnosed pulmonary arterial hypertension. Eur Heart J Digit Health 3(2):265–275 36713008 10.1093/ehjdh/ztac022 PMC9708011

[CR7] Alkhodari M, Widatalla N, Wahbah M, Al Sakaji R, Funamoto K, Krishnan A, Kimura Y, Khandoker AH (2022) Deep learning identifies cardiac coupling between mother and fetus during gestation. Front Cardiovasc Med 9:926965 35966548 10.3389/fcvm.2022.926965 PMC9372367

[CR8] Anand A, Kadian T, Shetty MK, Gupta A (2022) Explainable ai decision model for ECG data of cardiac disorders. Biomed Signal Process Control 75:103584

[CR9] Angelaki E, Marketou ME, Barmparis GD, Patrianakos A, Vardas PE, Parthenakis F, Tsironis GP (2021) Detection of abnormal left ventricular geometry in patients without cardiovascular disease through machine learning: an ECG-based approach. J Clin Hypertens 23(5):935–945 10.1111/jch.14200 PMC8678829 33507615

[CR10] Apama C, Rohini P, Pandiyarasan V (2022) Interpretation of ResNet-50 model for MI related cardiac events using explainable grad-cam approach. In: Current directions in biomedical engineering, vol 8. De Gruyter, Berlin, pp 723–726

[CR11] Apley DW (2020) Zhu, J: Visualizing the effects of predictor variables in black box supervised learning models. J R Stat Soc Ser B Stat Methodol 82(4):1059–1086

[CR12] Arrieta AB, Díaz-Rodríguez N, Del Ser J, Bennetot A, Tabik S, Barbado A, García S, Gil-López S, Molina D, Benjamins R et al (2020) Explainable artificial intelligence (XAI): concepts, taxonomies, opportunities and challenges toward responsible AI. Inf Fusion 58:82–115

[CR13] Attia ZI, Lerman G, Friedman PA (2021) Deep neural networks learn by using human-selected electrocardiogram features and novel features. Eur Heart J Digit Health 2(3):446–455 36713603 10.1093/ehjdh/ztab060 PMC9707937

[CR14] Aufiero S, Bleijendaal H, Robyns T, Vandenberk B, Krijger C, Bezzina C, Zwinderman AH, Wilde AA, Pinto YM (2022) A deep learning approach identifies new ECG features in congenital long QT syndrome. BMC Med 20(1):1–12 35501785 10.1186/s12916-022-02350-z PMC9063181

[CR15] Bach S, Binder A, Montavon G, Klauschen F, Müller K-R, Samek W (2015) On pixel-wise explanations for non-linear classifier decisions by layer-wise relevance propagation. PLoS ONE 10(7):0130140 10.1371/journal.pone.0130140 PMC4498753 26161953

[CR16] Bacoyannis T, Ly B, Cedilnik N, Cochet H, Sermesant M (2021) Deep learning formulation of electrocardiographic imaging integrating image and signal information with data-driven regularization. EP Europace 23(Supplement-1):55–62 10.1093/europace/euaa391 33751073

[CR17] Bahani K, Moujabbir M, Ramdani M (2021) An accurate fuzzy rule-based classification systems for heart disease diagnosis. Sci Afr 14:01019

[CR18] Bahrami N, Retson T, Blansit K, Wang K, Hsiao A (2019) Automated selection of myocardial inversion time with a convolutional neural network: spatial temporal ensemble myocardium inversion network (STEMI-Net). Magn Reson Med 81(5):3283–3291 30714197 10.1002/mrm.27680 PMC7962153

[CR19] Beetz M, Banerjee A, Grau V (2022) Multi-domain variational autoencoders for combined modeling of MRI-based biventricular anatomy and ECG-based cardiac electrophysiology. Front Physiol 13:886723 35755443 10.3389/fphys.2022.886723 PMC9213788

[CR20] Beetz M, Corral Acero J, Banerjee A, Eitel I, Zacur E, Lange T, Stiermaier T, Evertz R, Backhaus SJ, Thiele H et al (2022) Interpretable cardiac anatomy modeling using variational mesh autoencoders. Front Cardiovasc Med 9:983868 36620629 10.3389/fcvm.2022.983868 PMC9813669

[CR21] Bhardwaj A, Singh S, Joshi D (2023) Explainable deep convolutional neural network for valvular heart diseases classification using pcg signals. IEEE Trans Instrum Meas. 10.1109/TIM.2023.3274174

[CR22] Biffi C, Oktay O, Tarroni G, Bai W, De Marvao A, Doumou G, Rajchl M, Bedair R, Prasad S, Cook S et al (2018) Learning interpretable anatomical features through deep generative models: application to cardiac remodeling. In: Medical image computing and computer assisted intervention—MICCAI 2018: 21st international conference, Granada, Spain, 16–20 September 2018, Proceedings, Part II 11. Springer, Cham, pp 464–471

[CR23] Bodini M, Rivolta MW, Sassi R (2021) Opening the black box: interpretability of machine learning algorithms in electrocardiography. Philos Trans R Soc A 379(2212):20200253 10.1098/rsta.2020.0253 34689625

[CR24] Brisimi TS, Xu T, Wang T, Dai W, Adams WG, Paschalidis IC (2018) Predicting chronic disease hospitalizations from electronic health records: an interpretable classification approach. Proc IEEE 106(4):690–707 10.1109/JPROC.2017.2789319 PMC6419763 30886441

[CR57] British Heart Foundation B (2023) Global Heart & Circulatory Diseases Factsheet. https://www.bhf.org.uk/

[CR25] Bruijn H, Warnier M, Janssen M (2022) The perils and pitfalls of explainable AI: strategies for explaining algorithmic decision-making. Gov Inf Q 39(2):101666

[CR26] Cao Y, Liu W, Zhang S, Xu L, Zhu B, Cui H, Geng N, Han H, Greenwald SE (2022) Detection and localization of myocardial infarction based on multi-scale ResNet and attention mechanism. Front Physiol 13:24 10.3389/fphys.2022.783184 PMC8832050 35153827

[CR27] Cetin I, Stephens M, Camara O, Ballester MAG (2023) Attri-VAE: attribute-based interpretable representations of medical images with variational autoencoders. Comput Med Imaging Graph 104:102158 36638626 10.1016/j.compmedimag.2022.102158

[CR28] Chaddad A, Peng J, Xu J, Bouridane A (2023) Survey of explainable AI techniques in healthcare. Sensors 23(2):634 36679430 10.3390/s23020634 PMC9862413

[CR29] Chalasani P, Chen J, Chowdhury AR, Wu X, Jha S (2020) Concise explanations of neural networks using adversarial training. In: International conference on machine learning. PMLR, pp 1383–1391

[CR30] Chattopadhay A, Sarkar A, Howlader P, Balasubramanian VN (2018) Grad-CAM++: generalized gradient-based visual explanations for deep convolutional networks. In: 2018 IEEE Winter conference on applications of computer vision (WACV). IEEE, pp 839–847

[CR31] Chen P, Dong W, Wang J, Lu X, Kaymak U, Huang Z (2020) Interpretable clinical prediction via attention-based neural network. BMC Med Inform Decis Mak 20(3):1–9 32646437 10.1186/s12911-020-1110-7 PMC7346336

[CR32] Chen H, Gomez C, Huang C-M, Unberath M (2022) Explainable medical imaging AI needs human-centered design: guidelines and evidence from a systematic review. npj Digit Med 5(1):156 36261476 10.1038/s41746-022-00699-2 PMC9581990

[CR33] Chen S, Hu W, Yang Y, Cai J, Luo Y, Gong L, Li Y, Si A, Zhang Y, Liu S et al (2023a) Predicting six-month re-admission risk in heart failure patients using multiple machine learning methods: a study based on the Chinese heart failure population database. J Clin Med 12(3):870 36769515 10.3390/jcm12030870 PMC9918116

[CR34] Chen L, Fu G, Jiang C (2023b) Deep learning-derived 12-lead electrocardiogram-based genotype prediction for hypertrophic cardiomyopathy: a pilot study. Ann Med 55(1):2235564 37467172 10.1080/07853890.2023.2235564 PMC10360981

[CR35] Chen C, Zhao HY, Zheng SH, Ramachandra RA, He X, Zhang YH, Sudarshan VK (2023b) Interpretable hybrid model for an automated patient-wise categorization of hypertensive and normotensive electrocardiogram signals. Comput Methods Programs Biomed Update 3:100097

[CR36] Cho J, Lee B, Kwon J-M, Lee Y, Park H, Oh B-H, Jeon K-H, Park J, Kim K-H (2021) Artificial intelligence algorithm for screening heart failure with reduced ejection fraction using electrocardiography. ASAIO J 67(3):314–321 33627606 10.1097/MAT.0000000000001218

[CR37] Choi BS, Yoo SK, Moon J, Chung SY, Oh J, Baek S, Kim Y, Chang JS, Kim H, Kim JS (2023) Acute coronary event (ACE) prediction following breast radiotherapy by features extracted from 3D, CT, dose, and cardiac structures. Med Phys 50(10):6409–6420 10.1002/mp.16398 36974390

[CR38] Chou Y-L, Moreira C, Bruza P, Ouyang C, Jorge J (2022) Counterfactuals and causability in explainable artificial intelligence: theory, algorithms, and applications. Inf Fusion 81:59–83

[CR39] Clough JR, Oksuz I, Puyol-Antón E, Ruijsink B, King AP, Schnabel JA (2019) Global and local interpretability for cardiac MRI classification. In: International conference on medical image computing and computer-assisted intervention. Springer, Cham, pp 656–664

[CR40] Commandeur F, Slomka PJ, Goeller M, Chen X, Cadet S, Razipour A, McElhinney P, Gransar H, Cantu S, Miller RJ et al (2020) Machine learning to predict the long-term risk of myocardial infarction and cardiac death based on clinical risk, coronary calcium, and epicardial adipose tissue: a prospective study. Cardiovasc Res 116(14):2216–2225 31853543 10.1093/cvr/cvz321 PMC7750990

[CR41] Cui X, Lee JM, Hsieh J (2019) An integrative 3C evaluation framework for explainable artificial intelligence. In: Twenty-fifth Americas conference on information systems, Cancun

[CR42] Dakshit S, Maweu BM, Dakshit S, Prabhakaran B (2022) Core-set selection using metrics-based explanations (CSUME) for multiclass ECG. In: 2022 IEEE 10th international conference on healthcare informatics (ICHI). IEEE, pp 217–225

[CR43] Decoodt P, Liang TJ, Bopardikar S, Santhanam H, Eyembe A, Garcia-Zapirain B, Sierra-Sosa D (2023) Hybrid classical-quantum transfer learning for cardiomegaly detection in chest X-rays. J Imaging 9(7):128 37504805 10.3390/jimaging9070128 PMC10381726

[CR44] DeYoung J, Jain S, Rajani NF, Lehman E, Xiong C, Socher R, Wallace BC (2019) Eraser: a benchmark to evaluate rationalized NLP models. arXiv preprint. arXiv:1911.03429

[CR45] Di Martino F, Delmastro F (2022) Explainable ai for clinical and remote health applications: a survey on tabular and time series data. Artif Intell Rev 56:5261–5315 10.1007/s10462-022-10304-3 PMC9607788 36320613

[CR46] Diaz Ochoa JG, Maier L, Csiszar O (2023) Bayesian logical neural networks for human-centered applications in medicine. Front Bioinform 3:1082941 36875147 10.3389/fbinf.2023.1082941 PMC9975151

[CR47] Ding Z, Chen G, Zhang L, Baheti B, Wu R, Liao W, Liu X, Hou J, Mao Z, Guo Y et al (2023) Residential greenness and cardiac conduction abnormalities: epidemiological evidence and an explainable machine learning modeling study. Chemosphere 339:139671 10.1016/j.chemosphere.2023.139671 37517666

[CR48] Doborjeh M, Doborjeh Z, Merkin A, Krishnamurthi R, Enayatollahi R, Feigin V, Kasabov N (2022) Personalised spiking neural network models of clinical and environmental factors to predict stroke. Cogn Comput 14:2187–2202

[CR49] Dong T, Sinha S, Zhai B, Fudulu DP, Chan J, Narayan P, Judge A, Caputo M, Dimagli A, Benedetto U et al (2023) Cardiac surgery risk prediction using ensemble machine learning to incorporate legacy risk scores: a benchmarking study. Digit Health 9:20552076231187604 37492033 10.1177/20552076231187605 PMC10363892

[CR50] Doshi-Velez F, Kim B (2017) Towards a rigorous science of interpretable machine learning. arXiv preprint. arXiv:1702.08608

[CR51] Duffy G, Jain I, He B, Ouyang D (2021) Interpretable deep learning prediction of 3D assessment of cardiac function. In: Pacific symposium on biocomputing 2022. World Scientific, Singapore, pp 231–241 34890152

[CR52] Duval A, Nogueira D, Dissler N, Maskani Filali M, Delestro Matos F, Chansel-Debordeaux L, Ferrer-Buitrago M, Ferrer E, Antequera V, Ruiz-Jorro M et al (2023) A hybrid artificial intelligence model leverages multi-centric clinical data to improve fetal heart rate pregnancy prediction across time-lapse systems. Hum Reprod 38(4):596–608 36763673 10.1093/humrep/dead023 PMC10068266

[CR53] Fan Y, Dong J, Wu Y, Shen M, Zhu S, He X, Jiang S, Shao J, Song C (2022) Development of machine learning models for mortality risk prediction after cardiac surgery. Cardiovasc Diagn Ther Diagn Ther 12(1):12 35282663 10.21037/cdt-21-648 PMC8898685

[CR54] Fang R, Lu C-C, Chuang C-T, Chang W-H (2022) A visually interpretable detection method combines 3-D ECG with a multi-VGG neural network for myocardial infarction identification. Comput Methods Programs Biomed 219:106762 35378394 10.1016/j.cmpb.2022.106762

[CR55] Fang H, Shi C, Chen C-H (2020) BioExpDNN: bioinformatic explainable deep neural network. In: 2020 IEEE International conference on bioinformatics and biomedicine (BIBM). IEEE, pp 2461–2467

[CR56] Forte JC, Yeshmagambetova G, Grinten ML, Scheeren TW, Nijsten MW, Mariani MA, Henning RH, Epema AH (2022) Comparison of machine learning models including preoperative, intraoperative, and postoperative data and mortality after cardiac surgery. JAMA Netw Open 5(10):2237970–2237970 10.1001/jamanetworkopen.2022.37970 PMC9606847 36287565

[CR58] Gandin I, Saccani S, Coser A, Scagnetto A, Cappelletto C, Candido R, Barbati G, Di Lenarda A (2023) Deep-learning-based prognostic modeling for incident heart failure in patients with diabetes using electronic health records: a retrospective cohort study. PLoS ONE 18(2):0281878 10.1371/journal.pone.0281878 PMC9943005 36809251

[CR59] Ganeshkumar M, Ravi V, Sowmya V, Gopalakrishnan E, Soman K (2021) Explainable deep learning-based approach for multilabel classification of electrocardiogram. IEEE Trans Eng Manag. 10.1109/TEM.2021.3104751

[CR60] Gao S, Zhou H, Gao Y, Zhuang X (2023) BayeSeg: Bayesian modeling for medical image segmentation with interpretable generalizability. arXiv preprint. arXiv:2303.01710 10.1016/j.media.2023.102889 37467643

[CR61] Gee AH, Garcia-Olano D, Ghosh J, Paydarfar D (2019) Explaining deep classification of time-series data with learned prototypes. In: CEUR workshop proceedings, vol 2429. NIH Public Access, p 15 PMC8050893 33867901

[CR62] Ghorbani A, Ouyang D, Abid A, He B, Chen JH, Harrington RA, Liang DH, Ashley EA, Zou JY (2020) Deep learning interpretation of echocardiograms. NPJ Digit Med 3(1):10 31993508 10.1038/s41746-019-0216-8 PMC6981156

[CR63] Gilpin LH, Bau D, Yuan BZ, Bajwa A, Specter M, Kagal L (2018) Explaining explanations: an overview of interpretability of machine learning. In: 2018 IEEE 5th International conference on data science and advanced analytics (DSAA). IEEE, pp 80–89

[CR64] Gkontra P, Quaglio G, Garmendia AT, Lekadir K (2023) Challenges of machine learning and AI (what is next?), responsible and ethical AI. In: Clinical applications of artificial intelligence in real-world data. Springer, Cham, p 263

[CR65] González S, Hsieh W-T, Burba D, Chen TP-C, Wang C-L, Wu VC-C, Chang S-H (2022) Interpretable estimation of the risk of heart failure hospitalization from a 30-second electrocardiogram. In: 2022 E-Health and bioengineering conference (EHB). IEEE, pp 1–4

[CR66] Goswami PP, Deshpande T, Rotake DR, Singh SG (2023) Near perfect classification of cardiac biomarker troponin-i in human serum assisted by SnS2-CNT composite, explainable ML, and operating-voltage-selection-algorithm. Biosens Bioelectron 220:114915 36403491 10.1016/j.bios.2022.114915

[CR67] Greenwell BM, Boehmke BC, McCarthy AJ (2018) A simple and effective model-based variable importance measure. arXiv preprint. arXiv:1805.04755

[CR68] Guleria P, Naga Srinivasu P, Ahmed S, Almusallam N, Alarfaj FK (2022) XAI framework for cardiovascular disease prediction using classification techniques. Electronics 11(24):4086

[CR69] Guo F, Ng M, Goubran M, Petersen SE, Piechnik SK, Neubauer S, Wright G (2020) Improving cardiac MRI convolutional neural network segmentation on small training datasets and dataset shift: a continuous kernel cut approach. Med Image Anal 61:101636 31972427 10.1016/j.media.2020.101636

[CR70] Halme H-L, Ihalainen T, Suomalainen O, Loimaala A, Mätzke S, Uusitalo V, Sipilä O, Hippeläinen E (2022) Convolutional neural networks for detection of transthyretin amyloidosis in 2D scintigraphy images. EJNMMI Res 12(1):1–11 10.1186/s13550-022-00897-9 PMC9079204 35524861

[CR71] Hamatani Y, Nishi H, Iguchi M, Esato M, Tsuji H, Wada H, Hasegawa K, Ogawa H, Abe M, Fukuda S et al (2022) Machine learning risk prediction for incident heart failure in patients with atrial fibrillation. JACC Asia 2(6):706–716 36444329 10.1016/j.jacasi.2022.07.007 PMC9700042

[CR72] Haque A, Stubbs D, Hubig NC, Spinale FG, Richardson WJ (2022) Interpretable machine learning predicts cardiac resynchronization therapy responses from personalized biochemical and biomechanical features. BMC Med Inform Decis Mak 22(1):282 36316772 10.1186/s12911-022-02015-0 PMC9620606

[CR73] Hase P, Bansal M (2020) Evaluating explainable AI: which algorithmic explanations help users predict model behavior? arXiv preprint. arXiv:2005.01831

[CR74] Herm L-V, Heinrich K, Wanner J, Janiesch C (2023) Stop ordering machine learning algorithms by their explainability! A user-centered investigation of performance and explainability. Int J Inf Manage 69:102538

[CR75] Ho ES, Ding Z (2022) Electrocardiogram analysis of post-stroke elderly people using one-dimensional convolutional neural network model with gradient-weighted class activation mapping. Artif Intell Med 130:102342 35809968 10.1016/j.artmed.2022.102342

[CR77] Hong S, Xiao C, Ma T, Li H, Sun J (2019) MINA: multilevel knowledge-guided attention for modeling electrocardiography signals. arXiv preprint. arXiv:1905.11333

[CR76] Hong L, Xu H, Ge C, Tao H, Shen X, Song X, Guan D, Zhang C (2022) Prediction of low cardiac output syndrome in patients following cardiac surgery using machine learning. Front Med 9:973147 10.3389/fmed.2022.973147 PMC9448978 36091676

[CR78] Hooker S, Erhan D, Kindermans P-J, Kim B (2019) A benchmark for interpretability methods in deep neural networks. In: Advances in neural information processing systems 32 (NeurIPS 2019)

[CR79] Hu L-H, Betancur J, Sharir T, Einstein AJ, Bokhari S, Fish MB, Ruddy TD, Kaufmann PA, Sinusas AJ, Miller EJ et al (2020) Machine learning predicts per-vessel early coronary revascularization after fast myocardial perfusion spect: results from multicentre refine spect registry. Eur Heart J Cardiovasc Imaging 21(5):549–559 31317178 10.1093/ehjci/jez177 PMC7167744

[CR80] Hu Y, Feng T, Wang M, Liu C, Tang H (2023) Detection of paroxysmal atrial fibrillation from dynamic ECG recordings based on a deep learning model. J Pers Med 13(5):820 37240990 10.3390/jpm13050820 PMC10220587

[CR81] Huang Z, Gan Y, Lye T, Liu Y, Zhang H, Laine A, Angelini E, Hendon C (2023) Cardiac adipose tissue segmentation via image-level annotations. IEEE J Biomed Health Inf. 10.1109/JBHI.2023.3263838 10.1109/JBHI.2023.3263838 PMC10349643 37023157

[CR82] Hur C, Wi J, Kim Y (2020) Facilitating the development of deep learning models with visual analytics for electronic health records. Int J Environ Res Public Health 17(22):8303 33182703 10.3390/ijerph17228303 PMC7697823

[CR83] Huynh J, Masoudi S, Noorbakhsh A, Mahmoodi A, Kligerman S, Yen A, Jacobs K, Hahn L, Hasenstab K, Pazzani M et al (2022) Deep learning radiographic assessment of pulmonary edema: optimizing clinical performance, training with serum biomarkers. IEEE Access 10:48577–48588

[CR84] Janik A, Dodd J, Ifrim G, Sankaran K, Curran K (2021) Interpretability of a deep learning model in the application of cardiac MRI segmentation with an ACDC challenge dataset. In: Medical imaging 2021: image processing, vol 11596. SPIE, pp 861–872

[CR85] Jekova I, Christov I, Krasteva V (2022) Atrioventricular synchronization for detection of atrial fibrillation and flutter in one to twelve ECG leads using a dense neural network classifier. Sensors 22(16):6071 36015834 10.3390/s22166071 PMC9413391

[CR86] Jiang M, Qiu Y, Zhang W, Zhang J, Wang Z, Ke W, Wu Y, Wang Z (2022) Visualization deep learning model for automatic arrhythmias classification. Physiol Meas 43(8):085003 10.1088/1361-6579/ac8469 35882225

[CR87] Jiao Y, Yuan J, Sodimu OM, Qiang Y, Ding Y (2022) Deep neural network-aided histopathological analysis of myocardial injury. Front Cardiovasc Med 8:724183 35083295 10.3389/fcvm.2021.724183 PMC8784602

[CR88] Jin Y, Liu J, Liu Y, Qin C, Li Z, Xiao D, Zhao L, Liu C (2021) A novel interpretable method based on dual-level attentional deep neural network for actual multilabel arrhythmia detection. IEEE Trans Instrum Meas 71:1–11

[CR89] Jin W, Li X, Fatehi M, Hamarneh G (2023a) Guidelines and evaluation of clinical explainable AI in medical image analysis. Med Image Anal 84:102684 36516555 10.1016/j.media.2022.102684

[CR90] Jin W, Li X, Hamarneh G (2023b) Rethinking ai explainability and plausibility. arXiv preprint. arXiv:2303.17707

[CR91] Johnson E, Mohan S, Gaudio A, Smailagic A, Faloutsos C, Campilho A (2022) Heartspot: privatized and explainable data compression for cardiomegaly detection. In: 2022 IEEE-EMBS international conference on biomedical and health informatics (BHI). IEEE, pp 01–04

[CR92] Jones Y, Deligianni F, Dalton J (2020) Improving ECG classification interpretability using saliency maps. In: 2020 IEEE 20th international conference on bioinformatics and bioengineering (BIBE). IEEE, pp 675–682

[CR93] Kakogeorgiou I, Karantzalos K (2021) Evaluating explainable artificial intelligence methods for multi-label deep learning classification tasks in remote sensing. Int J Appl Earth Obs Geoinf 103:102520

[CR94] Kan C, Ye Z, Zhou H, Cheruku SR (2023) DG-ECG: multi-stream deep graph learning for the recognition of disease-altered patterns in electrocardiogram. Biomed Signal Process Control 80:104388

[CR95] Karatzia L, Aung N, Aksentijevic D (2022) Artificial intelligence in cardiology: hope for the future and power for the present. Front Cardiovasc Med 9:945726 36312266 10.3389/fcvm.2022.945726 PMC9608631

[CR96] Karoui A, Bendahmane M, Zemzemi N (2021) Cardiac activation maps reconstruction: a comparative study between data-driven and physics-based methods. Front Physiol 12:686136 34512373 10.3389/fphys.2021.686136 PMC8428526

[CR97] Karri R, Kawai A, Thong YJ, Ramson DM, Perry LA, Segal R, Smith JA, Penny-Dimri JC (2021) Machine learning outperforms existing clinical scoring tools in the prediction of postoperative atrial fibrillation during intensive care unit admission after cardiac surgery. Heart Lung Circ 30(12):1929–1937 34215511 10.1016/j.hlc.2021.05.101

[CR98] Kawakami M, Karashima S, Morita K, Tada H, Okada H, Aono D, Kometani M, Nomura A, Demura M, Furukawa K et al (2022) Explainable machine learning for atrial fibrillation in the general population using a generalized additive model – a cross-sectional study. Circ Rep 4(2):73–82 35178483 10.1253/circrep.CR-21-0151 PMC8811230

[CR99] Khurshid S, Friedman S, Reeder C, Di Achille P, Diamant N, Singh P, Harrington LX, Wang X, Al-Alusi MA, Sarma G et al (2022) ECG-based deep learning and clinical risk factors to predict atrial fibrillation. Circulation 145(2):122–133 34743566 10.1161/CIRCULATIONAHA.121.057480 PMC8748400

[CR100] Killian MO, Tian S, Xing A, Hughes D, Gupta D, Wang X, He Z ( (2023) Prediction of outcomes after heart transplantation in pediatric patients using national registry data: evaluation of machine learning approaches. JMIR Cardio 7:45352 10.2196/45352 PMC10334720 37338974

[CR101] Kofler A, Pali M-C, Schaeffter T, Kolbitsch C (2023) Deep supervised dictionary learning by algorithm unrolling-application to fast 2D dynamic MR image reconstruction. Med Phys 50(5):2939–2960 36565150 10.1002/mp.16182

[CR102] Kogan E, Didden E-M, Lee E, Nnewihe A, Stamatiadis D, Mataraso S, Quinn D, Rosenberg D, Chehoud C, Bridges C (2023) A machine learning approach to identifying patients with pulmonary hypertension using real-world electronic health records. Int J Cardiol 374:95–99 36528138 10.1016/j.ijcard.2022.12.016

[CR103] Kor C-T, Li Y-R, Lin P-R, Lin S-H, Wang B-Y, Lin C-H (2022) Explainable machine learning model for predicting first-time acute exacerbation in patients with chronic obstructive pulmonary disease. J Pers Med 12(2):228 35207716 10.3390/jpm12020228 PMC8879653

[CR104] Kucukseymen S, Arafati A, Al-Otaibi T, El-Rewaidy H, Fahmy AS, Ngo LH, Nezafat R (2022) Noncontrast cardiac magnetic resonance imaging predictors of heart failure hospitalization in heart failure with preserved ejection fraction. J Magn Reson Imaging 55(6):1812–1825 34559435 10.1002/jmri.27932

[CR105] Kukar M, Kononenko I, Grošelj C (2011) Modern parameterization and explanation techniques in diagnostic decision support system: a case study in diagnostics of coronary artery disease. Artif Intell Med 52(2):77–90 21646000 10.1016/j.artmed.2011.04.009

[CR106] Kumarakulasinghe NB, Blomberg T, Liu J, Leao AS, Papapetrou P (2020) Evaluating local interpretable model-agnostic explanations on clinical machine learning classification models. In: 2020 IEEE 33rd International symposium on computer-based medical systems (CBMS). IEEE, pp 7–12

[CR107] Kuppa A, Le-Khac N-A (2020) Black box attacks on explainable artificial intelligence (XAI) methods in cyber security. In: 2020 international joint conference on neural networks (IJCNN). IEEE, pp 1–8

[CR108] Kwon BC, Choi M-J, Kim JT, Choi E, Kim YB, Kwon S, Sun J, Choo J (2018) RetainVis: visual analytics with interpretable and interactive recurrent neural networks on electronic medical records. IEEE Trans Vis Comput Graph 25(1):299–309 10.1109/TVCG.2018.2865027 30136973

[CR109] Kwon J, Kim K-H, Medina-Inojosa J, Jeon K-H, Park J, Oh B-H (2020) Artificial intelligence for early prediction of pulmonary hypertension using electrocardiography. J Heart Lung Transplant 39(8):805–814 32381339 10.1016/j.healun.2020.04.009

[CR110] Kwon J, Kim K-H, Eisen HJ, Cho Y, Jeon K-H, Lee SY, Park J, Oh B-H (2021) Artificial intelligence assessment for early detection of heart failure with preserved ejection fraction based on electrocardiographic features. Eur Heart J Digit Health 2(1):106–116 36711179 10.1093/ehjdh/ztaa015 PMC9707919

[CR111] Lagopoulos A, Hristu-Varsakelis D (2022) Measuring the left ventricular ejection fraction using geometric features. In: 2022 IEEE 35th International symposium on computer-based medical systems (CBMS). IEEE, pp 1–6

[CR112] Le KH, Pham HH, Nguyen TB, Nguyen TA, Thanh TN, Do CD (2023) LightX3ECG: a lightweight and explainable deep learning system for 3-lead electrocardiogram classification. Biomed Signal Process Control 85:104963

[CR114] Lee H, Shin M (2021) Learning explainable time-morphology patterns for automatic arrhythmia classification from short single-lead ECGS. Sensors 21(13):4331 34202805 10.3390/s21134331 PMC8272104

[CR113] Lee SH, Geng H, Arnold J, Caruana R, Fan Y, Rosen MA, Apte AP, Deasy JO, Bradley JD, Xiao Y (2023) Interpretable machine learning for choosing radiation dose-volume constraints on cardio-pulmonary substructures associated with overall survival in NRG oncology RTOG 0617. Int J Radiat Oncol Biol Phys 117(5):1270–1286 10.1016/j.ijrobp.2023.06.009 PMC10728350 37343707

[CR115] Leur RR, Taha K, Bos MN, Heijden JF, Gupta D, Cramer MJ, Hassink RJ, Harst P, Doevendans PA, Asselbergs FW et al (2021) Discovering and visualizing disease-specific electrocardiogram features using deep learning: proof-of-concept in phospholamban gene mutation carriers. Circ Arrhythm Electrophysiol 14(2):009056 10.1161/CIRCEP.120.009056 PMC7892204 33401921

[CR116] Leventi-Peetz A-M, Weber K (2022) Rashomon effect and consistency in explainable artificial intelligence (XAI). In: Proceedings of the future technologies conference (FTC) 2022, vol 1. Springer, Cham, pp 796–808

[CR117] Li R, Yin C, Yang S, Qian B, Zhang P (2020) Marrying medical domain knowledge with deep learning on electronic health records: a deep visual analytics approach. J Med Internet Res 22(9):20645 10.2196/20645 PMC7551124 32985996

[CR118] Li J, Liu S, Hu Y, Zhu L, Mao Y, Liu J (2022) Predicting mortality in intensive care unit patients with heart failure using an interpretable machine learning model: retrospective cohort study. J Med Internet Res 24(8):38082 10.2196/38082 PMC9399880 35943767

[CR119] Liang Y, Guo C (2023) Heart failure disease prediction and stratification with temporal electronic health records data using patient representation. Biocybern Biomed Eng 43(1):124–141

[CR120] Lin Y-C, Lee Y-C, Tsai W-C, Beh W-K, Wu A-YA (2020) Explainable deep neural network for identifying cardiac abnormalities using class activation map. In: 2020 Computing in cardiology. IEEE, pp 1–4

[CR121] Linardatos P, Papastefanopoulos V, Kotsiantis S (2020) Explainable AI: a review of machine learning interpretability methods. Entropy 23(1):18 33375658 10.3390/e23010018 PMC7824368

[CR122] Lindow T, Palencia-Lamela I, Schlegel TT, Ugander M (2022) Heart age estimated using explainable advanced electrocardiography. Sci Rep 12(1):9840 35701514 10.1038/s41598-022-13912-9 PMC9198017

[CR123] Lisboa PJ, Jayabalan M, Ortega-Martorell S, Olier I Medved D Nilsson J (2022) Enhanced survival prediction using explainable artificial intelligence in heart transplantation. Sci Rep 12(1):19525 10.1038/s41598-022-23817-2 PMC9663731 36376402

[CR124] Liu Z, Cao Q, Jin Q, Lin J, Lv G, Chen K (2023) Accurate detection of arrhythmias on raw electrocardiogram images: an aggregation attention multi-label model for diagnostic assistance. Med Eng Phys 114:103964 37030892 10.1016/j.medengphy.2023.103964

[CR125] Liu J, Yuan G, Yang C, Song H, Luo L (2023) An interpretable CNN for the segmentation of the left ventricle in cardiac MRI by real-time visualization. CMES Comput Model Eng Sci 135(2):1571–1587

[CR126] Lo Iacono F, Maragna R, Pontone G, Corino VD (2023) A robust radiomic-based machine learning approach to detect cardiac amyloidosis using cardiac computed tomography. Front Radiol 3:1193046 37588665 10.3389/fradi.2023.1193046 PMC10426499

[CR127] Loh HW, Ooi CP, Seoni S, Barua PD, Molinari F, Acharya UR (2022) Application of explainable artificial intelligence for healthcare: a systematic review of the last decade (2011–2022). Comput Methods Programs Biomed 226:107161 36228495 10.1016/j.cmpb.2022.107161

[CR128] Loncaric F, Castellote P-MM, Sanchez-Martinez S, Fabijanovic D, Nunno L, Mimbrero M, Sanchis L, Doltra A, Montserrat S, Cikes M et al (2021) Automated pattern recognition in whole-cardiac cycle echocardiographic data: capturing functional phenotypes with machine learning. J Am Soc Echocardiogr 34(11):1170–1183 34245826 10.1016/j.echo.2021.06.014

[CR129] Lopes RR, Bleijendaal H, Ramos LA, Verstraelen TE, Amin AS, Wilde AA, Pinto YM, Mol BA, Marquering HA (2021) Improving electrocardiogram-based detection of rare genetic heart disease using transfer learning: an application to phospholamban p.Arg14del mutation carriers. Comput Biol Med 131:104262 33607378 10.1016/j.compbiomed.2021.104262

[CR130] Lopes P, Silva E, Braga C, Oliveira T, Rosado L (2022) XAI systems evaluation: a review of human and computer-centred methods. Appl Sci 12(19):9423

[CR131] Lu S, Chen R, Wei W, Belovsky M, Lu X (2021) Understanding heart failure patients ehr clinical features via shap interpretation of tree-based machine learning model predictions. In: AMIA annual symposium proceedings, vol 2021. American Medical Informatics Association, Bethesda, p 813 PMC8861751 35308970

[CR132] Lundberg SM, Lee S-I (2017) A unified approach to interpreting model predictions. In: 31st Conference on neural information processing systems (NIPS 2017), Long Beach

[CR133] Ly B, Finsterbach S, Nuñez-Garcia M, Jaïs P, Garreau D, Cochet H, Sermesant M (2022) Interpretable prediction of post-infarct ventricular arrhythmia using graph convolutional network. In: International workshop on statistical atlases and computational models of the heart. Springer, Cham, pp 157–167

[CR134] Ma F, Wang Y, Xiao H, Yuan Y, Chitta R, Zhou J, Gao J (2019) Incorporating medical code descriptions for diagnosis prediction in healthcare. BMC Med Inform Decis Mak 19(6):1–13 31856806 10.1186/s12911-019-0961-2 PMC6921390

[CR135] Mahajan A, Esper S, Oo TH, McKibben J, Garver M, Artman J, Klahre C, Ryan J, Sadhasivam S, Holder-Murray J et al (2023) Development and validation of a machine learning model to identify patients before surgery at high risk for postoperative adverse events. JAMA Netw Open 6(7):2322285–2322285 10.1001/jamanetworkopen.2023.22285 PMC10329211 37418262

[CR136] Maiorana E, Romano C, Schena E, Massaroni C (2023) BIOWISH: biometric recognition using wearable inertial sensors detecting heart activity. IEEE Trans Depend Secure Comput. 10.1109/TDSC.2023.3268360 10.1109/TDSC.2023.3268360

[CR137] Makimoto H, Shiraga T, Kohlmann B, Magnisali CE, Gerguri S, Motoyama N, Clasen L, Bejinariu A, Klein K, Makimoto A et al (2022) Efficient screening for severe aortic valve stenosis using understandable artificial intelligence: a prospective diagnostic accuracy study. Eur Heart J Digit Health 3(2):141–152 36713014 10.1093/ehjdh/ztac029 PMC9707975

[CR138] Markov N, Ushenin K, Bozhko Y (2023) A convolutional recurrent model for the identification of patients with atrial fibrillation based on heart rate variability data during sinus rhythm. In: 2023 IEEE Ural-Siberian conference on biomedical engineering, radioelectronics and information technology (USBEREIT). IEEE, pp 072–075

[CR139] Melzi P, Tolosana R, Cecconi A, Sanz-Garcia A, Ortega GJ, Jimenez-Borreguero LJ, Vera-Rodriguez R (2021) Analyzing artificial intelligence systems for the prediction of atrial fibrillation from sinus-rhythm ECGs including demographics and feature visualization. Sci Rep 11(1):22786 34815461 10.1038/s41598-021-02179-1 PMC8610971

[CR140] Meng J, Xing R (2022) Inside the “black box’’: embedding clinical knowledge in data-driven machine learning for heart disease diagnosis. Cardiovasc Digit Health J 3(6):276–288 36589311 10.1016/j.cvdhj.2022.10.005 PMC9795264

[CR141] Michel P, Ngo N, Pons J-F, Delliaux S, Giorgi R (2021) A filter approach for feature selection in classification: application to automatic atrial fibrillation detection in electrocardiogram recordings. BMC Med Inform Decis Mak 21(4):1–17 33947379 10.1186/s12911-021-01427-8 PMC8094578

[CR142] Miran SM, Nelson SJ, Zeng-Treitler Q (2021) A model-agnostic approach for understanding heart failure risk factors. BMC Res Notes 14(1):184 34001210 10.1186/s13104-021-05596-7 PMC8130447

[CR143] Miranda E, Adiarto S, Bhatti FM, Zakiyyah AY, Aryuni M, Bernando C (2023) Understanding arteriosclerotic heart disease patients using electronic health records: a machine learning and shapley additive explanations approach. Healthc Inf Res 29(3):228–238 10.4258/hir.2023.29.3.228 PMC10440196 37591678

[CR144] Mohseni S, Zarei N, Ragan ED (2021) A multidisciplinary survey and framework for design and evaluation of explainable AI systems. ACM Trans Interact Intell Syst (TiiS) 11(3–4):1–45

[CR145] Mokhtari M, Tsang T, Abolmaesumi P, Liao R (2022) EchoGNN: explainable ejection fraction estimation with graph neural networks. In: International conference on medical image computing and computer-assisted intervention. Springer, Berlin, pp 360–369

[CR146] Molnar C, König G, Herbinger J, Freiesleben T, Dandl S, Scholbeck CA, Casalicchio G, Grosse-Wentrup M, Bischl B (2022) General pitfalls of model-agnostic interpretation methods for machine learning models. In: xxAI-beyond explainable AI: international workshop, held in conjunction with ICML 2020, 18 July 2020, Vienna, Austria, revised and extended papers. Springer, Cham, pp 39–68

[CR147] Montavon G, Lapuschkin S, Binder A, Samek W, Müller K-R (2017) Explaining nonlinear classification decisions with deep Taylor decomposition. Pattern Recogn 65:211–222

[CR148] Montavon G, Samek W, Müller K-R (2018) Methods for interpreting and understanding deep neural networks. Digit Signal Process 73:1–15

[CR149] Moreno-Sanchez PA (2020) Development of an explainable prediction model of heart failure survival by using ensemble trees. In: 2020 IEEE international conference on big data (Big Data). IEEE, pp 4902–4910

[CR150] Mousavi S, Afghah F, Acharya UR (2020) HAN-ECG: an interpretable atrial fibrillation detection model using hierarchical attention networks. Comput Biol Med 127:104057 33126126 10.1016/j.compbiomed.2020.104057 PMC7875017

[CR151] Nankani D, Baruah RD (2021) Ventricular arrhythmia classification and interpretation using residual neural network with guided backpropagation. In: TENCON 2021-2021 IEEE Region 10 conference (TENCON). IEEE, pp 574–579

[CR152] Nankani D, Baruah RD (2022) Atrial fibrillation classification and prediction explanation using transformer neural network. In: 2022 International joint conference on neural networks (IJCNN). IEEE, pp 01–08

[CR153] Nguyen MB, Dragulescu A, Chaturvedi R, Fan C-PS, Villemain O, Friedberg MK, Mertens LL (2022) Understanding complex interactions in pediatric diastolic function assessment. J Am Soc Echocardiogr 35(8):868–877 35341955 10.1016/j.echo.2022.03.017

[CR154] Niu J, Lu Y, Xu R, Fang F, Hong S, Huang L, Xue Y, Fei J, Zhang X, Zhou B et al (2023) The prognostic value of intraoperative HRV during anesthesia in patients presenting for non-cardiac surgery. BMC Anesthesiol 23(1):1–10 37161402 10.1186/s12871-023-02118-9 PMC10169477

[CR155] Novelli C, Taddeo M, Floridi L (2023) Accountability in artificial intelligence: what it is and how it works. AI Soc. 10.1007/s00146-023-01635-y 10.1007/s00146-023-01635-y

[CR156] Nurmaini S, Partan RU, Bernolian N, Sapitri AI, Tutuko B, Rachmatullah MN, Darmawahyuni A, Firdaus F, Mose JC (2022) Deep learning for improving the effectiveness of routine prenatal screening for major congenital heart diseases. J Clin Med 11(21):6454 36362685 10.3390/jcm11216454 PMC9653675

[CR157] Ogbomo-Harmitt S, Muffoletto M, Zeidan A, Qureshi A, King AP, Aslanidi O (2023) Exploring interpretability in deep learning prediction of successful ablation therapy for atrial fibrillation. Front Physiol 14:1054401 36998987 10.3389/fphys.2023.1054401 PMC10043207

[CR158] Oliveira M, Seringa J, Pinto FJ, Henriques R, Magalhães T (2023) Machine learning prediction of mortality in acute myocardial infarction. BMC Med Inform Decis Mak 23(1):1–16 37072766 10.1186/s12911-023-02168-6 PMC10111317

[CR159] Ovalle-Magallanes E, Avina-Cervantes JG, Cruz-Aceves I, Ruiz-Pinales J (2020) Transfer learning for stenosis detection in X-ray coronary angiography. Mathematics 8(9):1510

[CR160] Painchaud N, Duchateau N, Bernard O, Jodoin P-M (2022) Echocardiography segmentation with enforced temporal consistency. IEEE Trans Med Imaging 41(10):2867–2878 35533176 10.1109/TMI.2022.3173669

[CR161] Panicacci S, Donati M, Fanucci L, Bellini I, Profili F, Francesconi P (2019) Exploring machine learning algorithms to identify heart failure patients: the Tuscany region case study. In: 2019 IEEE 32nd International symposium on computer-based medical systems (CBMS). IEEE, pp 417–422

[CR162] Patel J, Ladani A, Sambamoorthi N, LeMasters T, Dwibedi N, Sambamoorthi U (2021) Predictors of co-occurring cardiovascular and gastrointestinal disorders among elderly with osteoarthritis. Osteoarthritis Cartilage Open 3(2):100148 36474979 10.1016/j.ocarto.2021.100148 PMC9718072

[CR163] Peng S, Huang J, Liu X, Deng J, Sun C, Tang J, Chen H, Cao W, Wang W, Duan X et al (2022) Interpretable machine learning for 28-day all-cause in-hospital mortality prediction in critically ill patients with heart failure combined with hypertension: a retrospective cohort study based on medical information Mart for intensive care database-IV and EICU databases. Front Cardiovasc Med 9:994359 36312291 10.3389/fcvm.2022.994359 PMC9597462

[CR164] Pérez-Pelegrí M, Monmeneu JV, López-Lereu MP, Pérez-Pelegrí L, Maceira AM, Bodí V, Moratal D (2021) Automatic left ventricle volume calculation with explainability through a deep learning weak-supervision methodology. Comput Methods Programs Biomed 208:106275 34274609 10.1016/j.cmpb.2021.106275

[CR165] Petersen SE, Matthews PM, Francis JM, Robson MD, Zemrak F, Boubertakh R, Young AA, Hudson S, Weale P, Garratt S et al (2015) UK biobank’s cardiovascular magnetic resonance protocol. J Cardiovasc Magn Reson 18(1):1–7 10.1186/s12968-016-0227-4 PMC4736703 26830817

[CR166] Pham T-H, Yin C, Mehta L, Zhang X, Zhang P (2023) A fair and interpretable network for clinical risk prediction: a regularized multi-view multi-task learning approach. Knowl Inf Syst 65(4):1487–1521 10.1007/s10115-022-01813-2 PMC10046420 36998311

[CR167] Pičulin M, Smole T, Žunkovič B, Kokalj E, Robnik-Šikonja M, Kukar M, Fotiadis DI, Pezoulas VC, Tachos NS, Barlocco F et al (2022) Disease progression of hypertrophic cardiomyopathy: modeling using machine learning. JMIR Med Inform 10(2):30483 10.2196/30483 PMC8851344 35107432

[CR168] Pieszko K, Shanbhag AD, Singh A, Hauser MT, Miller RJ, Liang JX, Motwani M, Kwieciński J, Sharir T, Einstein AJ et al (2023) Time and event-specific deep learning for personalized risk assessment after cardiac perfusion imaging. NPJ Digit Med 6(1):78 37127660 10.1038/s41746-023-00806-x PMC10151323

[CR169] Plumb G, Molitor D, Talwalkar AS (2018) Model agnostic supervised local explanations. In: Advances in neural information processing systems 31 (NeurIPS 2018)

[CR170] Portella JJ, Andonian BJ, Brown DE, Mansur J, Wales D, West VL, Kraus WE, Hammond WE (2022) Using machine learning to identify organ system specific limitations to exercise via cardiopulmonary exercise testing. IEEE J Biomed Health Inform 26(8):4228–4237 35353709 10.1109/JBHI.2022.3163402 PMC9512518

[CR171] Prifti E, Fall A, Davogustto G, Pulini A, Denjoy I, Funck-Brentano C, Khan Y, Durand-Salmon A, Badilini F, Wells QS et al (2021) Deep learning analysis of electrocardiogram for risk prediction of drug-induced arrhythmias and diagnosis of long QT syndrome. Eur Heart J 42(38):3948–3961 34468739 10.1093/eurheartj/ehab588

[CR172] Qu Y, Deng X, Lin S, Han F, Chang HH, Ou Y, Nie Z, Mai J, Wang X, Gao X et al (2022) Using innovative machine learning methods to screen and identify predictors of congenital heart diseases. Front Cardiovasc Med 8:797002 35071361 10.3389/fcvm.2021.797002 PMC8777022

[CR173] Ragnarsdottir H, Manduchi L, Michel H, Laumer F, Wellmann S, Ozkan E, Vogt JE (2022) Interpretable prediction of pulmonary hypertension in newborns using echocardiograms. In: DAGM German conference on pattern recognition. Springer, Cham, pp 529–542

[CR174] Rao S, Li Y, Ramakrishnan R, Hassaine A, Canoy D, Cleland J, Lukasiewicz T, Salimi-Khorshidi G, Rahimi K (2022) An explainable transformer-based deep learning model for the prediction of incident heart failure. IEEE J Biomed Health Inform 26(7):3362–3372 35130176 10.1109/JBHI.2022.3148820

[CR175] Rashed-Al-Mahfuz M, Moni MA, Lio’ P, Islam SMS, Berkovsky S, Khushi M, Quinn JM (2021) Deep convolutional neural networks based ECG beats classification to diagnose cardiovascular conditions. Biomed Eng Lett 11:147–162 34150350 10.1007/s13534-021-00185-w PMC8155180

[CR176] Rauf A, Ullah A, Rathi U, Ashfaq Z, Ullah H, Ashraf A, Kumar J, Faraz M, Akhtar W, Mehmoodi A et al (2023) Predicting stroke and mortality in mitral stenosis with atrial flutter: a machine learning approach. Ann Noninvasive Electrocardiol 28(5):e13078 37545120 10.1111/anec.13078 PMC10475890

[CR177] Ribeiro MT, Singh S, Guestrin C (2016) “Why should i trust you?” Explaining the predictions of any classifier. In: Proceedings of the 22nd ACM SIGKDD international conference on knowledge discovery and data mining, pp 1135–1144

[CR178] Ronneberger O, Fischer P, Brox T (2015) U-Net: convolutional networks for biomedical image segmentation. In: Medical image computing and computer-assisted intervention—MICCAI 2015: 18th international conference, Munich, Germany, 5–9 October 2015, Proceedings, Part III 18. Springer, Cham, pp 234–241

[CR179] Roseiro M, Henriques J, Paredes S, Rocha T, Sousa J (2023) An interpretable machine learning approach to estimate the influence of inflammation biomarkers on cardiovascular risk assessment. Comput Methods Programs Biomed 230:107347 36645940 10.1016/j.cmpb.2023.107347

[CR180] Rouhi R, Clausel M, Oster J, Lauer F (2021) An interpretable hand-crafted feature-based model for atrial fibrillation detection. Front Physiol 12:657304 34054575 10.3389/fphys.2021.657304 PMC8155476

[CR181] Rueda C, Rodríguez-Collado A, Fernández I, Canedo C, Ugarte MD, Larriba Y (2022) A unique cardiac electrocardiographic 3D model. Toward interpretable AI diagnosis. iScience 25(12):105617 36465104 10.1016/j.isci.2022.105617 PMC9712771

[CR182] Sager S, Bernhardt F, Kehrle F, Merkert M, Potschka A, Meder B, Katus H, Scholz E (2021) Expert-enhanced machine learning for cardiac arrhythmia classification. PLoS ONE 16(12):0261571 10.1371/journal.pone.0261571 PMC8699667 34941897

[CR183] Saito Y, Omae Y, Fukamachi D, Nagashima K, Mizobuchi S, Kakimoto Y, Toyotani J, Okumura Y (2022) Quantitative estimation of pulmonary artery wedge pressure from chest radiographs by a regression convolutional neural network. Heart Vessels 37(8):1387–1394 35220466 10.1007/s00380-022-02043-w PMC9239946

[CR184] Sakai A, Komatsu M, Komatsu R, Matsuoka R, Yasutomi S, Dozen A, Shozu K, Arakaki T, Machino H, Asada K et al (2022) Medical professional enhancement using explainable artificial intelligence in fetal cardiac ultrasound screening. Biomedicines 10(3):551 35327353 10.3390/biomedicines10030551 PMC8945208

[CR188] Salih A, Galazzo IB, Cruciani F, Brusini L, Radeva P (2022) Investigating explainable artificial intelligence for mri-based classification of dementia: a new stability criterion for explainable methods. In: 2022 IEEE International Conference on Image Processing (ICIP), pp. 4003–4007. IEEE

[CR186] Salih AM, Pujadas ER, Campello VM, McCracken C, Harvey NC, Neubauer S, Lekadir K, Nichols TE, Petersen SE, Raisi-Estabragh Z (2023a) Image-based biological heart age estimation reveals differential aging patterns across cardiac chambers. J Magn Reson Imaging 58(6):1797–1812 36929232 10.1002/jmri.28675 PMC10947470

[CR187] Salih A, Boscolo Galazzo I, Gkontra P, Lee AM, Lekadir K, Raisi-Estabragh Z, Petersen SE (2023b) Explainable artificial intelligence and cardiac imaging: toward more interpretable models. Circ Cardiovasc Imaging 16(4):e014519 37042240 10.1161/CIRCIMAGING.122.014519

[CR185] Salih AM, Galazzo IB, Raisi-Estabragh Z, Petersen SE, Menegaz G, Radeva P (2024) Characterizing the contribution of dependent features in XAI methods. IEEE J Biomed Health Inform. 10.1109/JBHI.2024.3395289 10.1109/JBHI.2024.3395289 38696291

[CR189] Sammani A, Leur RR, Henkens MT, Meine M, Loh P, Hassink RJ, Oberski DL, Heymans SR, Doevendans PA, Asselbergs FW et al (2022) Life-threatening ventricular arrhythmia prediction in patients with dilated cardiomyopathy using explainable electrocardiogram-based deep neural networks. Europace 24(10):1645–1654 35762524 10.1093/europace/euac054 PMC9559909

[CR190] Sang Y, Beetz M, Grau V (2022) Generation of 12-lead electrocardiogram with subject-specific, image-derived characteristics using a conditional variational autoencoder. In: 2022 IEEE 19th International symposium on biomedical imaging (ISBI). IEEE, pp 1–5

[CR191] Sangha V, Nargesi AA, Dhingra LS, Khunte A, Mortazavi BJ, Ribeiro AH, Banina E, Adeola O, Garg N, Brandt CA et al (2022) Detection of left ventricular systolic dysfunction from electrocardiographic images. Circulation. 10.1161/CIRCULATIONAHA.122.06264 10.1161/CIRCULATIONAHA.122.06264 PMC10982757 37489538

[CR192] Sangroya A, Jain S, Vig L, Anantaram C, Ukil A, Khandelwal S (2022) Generating conceptual explanations for DL based ECG classification model. In: The International FLAIRS conference proceedings, vol 35

[CR193] Sanjeevi G, Gopalakrishnan U, Pathinarupothi RK, Madathil T (2023) Automatic diagnostic tool for detection of regional wall motion abnormality from echocardiogram. J Med Syst 47(1):13 36700970 10.1007/s10916-023-01911-w

[CR194] Sannino G, De Pietro G, De Falco I (2021) Automatic extraction of interpretable knowledge to predict the survival of patients with heart failure. In: 2021 IEEE/ACM conference on connected health: applications, systems and engineering technologies (CHASE). IEEE, pp 166–173

[CR195] Sawano S, Kodera S, Katsushika S, Nakamoto M, Ninomiya K, Shinohara H, Higashikuni Y, Nakanishi K, Nakao T, Seki T et al (2022) Deep learning model to detect significant aortic regurgitation using electrocardiography. J Cardiol 79(3):334–341 34544652 10.1016/j.jjcc.2021.08.029

[CR196] Sbrollini A, Leoni C, Jongh MC, Morettini M, Burattini L, Swenne CA (2022) Feature contributions to ECG-based heart-failure detection: deep learning vs. statistical analysis. In: 2022 Computing in cardiology (CinC), vol 498. IEEE, pp 1–4

[CR197] Schrutka L, Anner P, Agibetov A, Seirer B, Dusik F, Rettl R, Duca F, Dalos D, Dachs T-M, Binder C et al (2022) Machine learning-derived electrocardiographic algorithm for the detection of cardiac amyloidosis. Heart 108(14):1137–1147 34716183 10.1136/heartjnl-2021-319846 PMC9240336

[CR198] Selvaraju RR, Cogswell M, Das A, Vedantam R, Parikh D, Batra D (2017) Grad-CAM: visual explanations from deep networks via gradient-based localization. In: Proceedings of the IEEE international conference on computer vision, pp 618–626

[CR199] Shah B, Kunal S, Bansal A, Jain J, Poundrik S, Shetty MK, Batra V, Chaturvedi V, Yusuf J, Mukhopadhyay S et al (2022) Heart rate variability as a marker of cardiovascular dysautonomia in post-covid-19 syndrome using artificial intelligence. Indian Pacing Electrophysiol J 22(2):70–76 35101582 10.1016/j.ipej.2022.01.004 PMC8800539

[CR200] Sharma Y, Coronato N, Brown DE (2022) Encoding cardiopulmonary exercise testing time series as images for classification using convolutional neural network. In: 2022 44th annual international conference of the IEEE Engineering in Medicine & Biology Society (EMBC). IEEE, pp 1611–1614 10.1109/EMBC48229.2022.9871878 PMC10436355 36086506

[CR203] Shi Z, Zeng G, Zhang L, Zhuang X, Li L, Yang G, Zheng G (2018) Bayesian voxdrn: A probabilistic deep voxelwise dilated residual network for whole heart segmentation from 3d mr images. In: Medical Image Computing and Computer Assisted Intervention–MICCAI 2018: 21st International Conference, Granada, Spain, September 16-20, 2018, Proceedings, Part IV 11, pp. 569–577. Springer

[CR201] Shi H, Yang D, Tang K, Hu C, Li L, Zhang L, Gong T, Cui Y (2022) Explainable machine learning model for predicting the occurrence of postoperative malnutrition in children with congenital heart disease. Clin Nutr 41(1):202–210 34906845 10.1016/j.clnu.2021.11.006

[CR202] Shin SJ, Park J, Lee S-H, Yang K, Park RW (2021) Predictability of mortality in patients with myocardial injury after noncardiac surgery based on perioperative factors via machine learning: retrospective study. JMIR Med Inform 9(10):32771 10.2196/32771 PMC8554678 34647900

[CR204] Silva A, Schrum M, Hedlund-Botti E, Gopalan N, Gombolay M (2023) Explainable artificial intelligence: evaluating the objective and subjective impacts of XAI on human–agent interaction. Int J Hum Comput Interact 39(7):1390–1404

[CR205] Singh P, Sharma A (2022) Interpretation and classification of arrhythmia using deep convolutional network. IEEE Trans Instrum Meas 71:1–12

[CR206] Singh A, Kwiecinski J, Miller RJ, Otaki Y, Kavanagh PB, Van Kriekinge SD, Parekh T, Gransar H, Pieszko K, Killekar A et al (2022) Deep learning for explainable estimation of mortality risk from myocardial positron emission tomography images. Circ Cardiovasc Imaging 15(9):014526 10.1161/CIRCIMAGING.122.014526 PMC10035936 36126124

[CR207] Singh A, Miller RJ, Otaki Y, Kavanagh P, Hauser MT, Tzolos E, Kwiecinski J, Van Kriekinge S, Wei C-C, Sharir T et al (2023) Direct risk assessment from myocardial perfusion imaging using explainable deep learning. Cardiovasc Imaging 16(2):209–220 10.1016/j.jcmg.2022.07.017 PMC10980287 36274041

[CR208] Slack D, Hilgard S, Jia E, Singh S, Lakkaraju H (2020) Fooling lime and shap: Adversarial attacks on post hoc explanation methods. In: Proceedings of the AAAI/ACM conference on AI, ethics, and society, pp 180–186

[CR209] Smole T, Žunkovič B, Pičulin M, Kokalj E, Robnik-Šikonja M, Kukar M, Fotiadis DI, Pezoulas VC, Tachos NS, Barlocco F et al (2021) A machine learning-based risk stratification model for ventricular tachycardia and heart failure in hypertrophic cardiomyopathy. Comput Biol Med 135:104648 34280775 10.1016/j.compbiomed.2021.104648

[CR210] Soares E, Angelov P, Gu X (2020) Autonomous learning multiple-model zero-order classifier for heart sound classification. Appl Soft Comput 94:106449

[CR211] Soto JT, Weston Hughes J, Sanchez PA, Perez M, Ouyang D, Ashley EA (2022) Multimodal deep learning enhances diagnostic precision in left ventricular hypertrophy. Eur Heart J Digit Health 3(3):380–389 36712167 10.1093/ehjdh/ztac033 PMC9707995

[CR212] Springenberg JT, Dosovitskiy A, Brox T, Riedmiller M (2014) Striving for simplicity: the all convolutional net. arXiv preprint. arXiv:1412.6806

[CR213] Stabellini N, Dmukauskas M, Bittencourt MS, Cullen J, Barda AJ, Moore JX, Dent S, Abdel-Qadir H, Kawatkar AA, Pandey A et al (2023) Social determinants of health and racial disparities in cardiac events in breast cancer. J Natl Compr Canc Netw 21(7):705–714 37433439 10.6004/jnccn.2023.7023

[CR216] Sun J, Darbehani F, Zaidi M, Wang B (2020) SAUNet: shape attentive U-Net for interpretable medical image segmentation. In: Medical image computing and computer assisted intervention—MICCAI 2020: 23rd international conference, Lima, Peru, 4–8 October 2020, Proceedings, Part IV 23. Springer, pp 797–806

[CR214] Sun R, Wang X, Jiang H, Yan Y, Dong Y, Yan W, Luo X, Miu H, Qi L, Huang Z (2022) Prediction of 30-day mortality in heart failure patients with hypoxic hepatitis: Development and external validation of an interpretable machine learning model. Front Cardiovasc Med 9:1035675 36386374 10.3389/fcvm.2022.1035675 PMC9649827

[CR215] Sundararajan M, Taly A, Yan Q (2017) Axiomatic attribution for deep networks. In: International conference on machine learning. PMLR, pp 3319–3328

[CR217] Szabo L, Raisi-Estabrag Z, Salih A, McCracken C, Pujadas ER, Gkontra P, Kiss M, Maurovich-Horvath P, Vago H, Merkely B et al (2022) Clinician’s guide to trustworthy and responsible artificial intelligence in cardiovascular imaging. Front Cardiovasc Med 9:1016032 36426221 10.3389/fcvm.2022.1016032 PMC9681217

[CR218] Tamarappoo BK, Lin A, Commandeur F, McElhinney PA, Cadet S, Goeller M, Razipour A, Chen X, Gransar H, Cantu S et al (2021) Machine learning integration of circulating and imaging biomarkers for explainable patient-specific prediction of cardiac events: a prospective study. Atherosclerosis 318:76–82 33239189 10.1016/j.atherosclerosis.2020.11.008 PMC7856265

[CR219] Tang Q, Cen X, Pan C (2022) Explainable and efficient deep early warning system for cardiac arrest prediction from electronic health records. Math Biosci Eng 19(10):9825–9841 36031970 10.3934/mbe.2022457

[CR220] Tong Q, Li C, Si W, Liao X, Tong Y, Yuan Z, Heng PA (2019) RIANet: recurrent interleaved attention network for cardiac mri segmentation. Comput Biol Med 109:290–302 31100582 10.1016/j.compbiomed.2019.04.042

[CR221] Tsuji T, Hirata Y, Kusunose K, Sata M, Kumagai S, Shiraishi K, Kotoku J (2023) Classification of chest X-ray images by incorporation of medical domain knowledge into operation branch networks. BMC Med Imaging 23(1):1–18 37161392 10.1186/s12880-023-01019-0 PMC10169130

[CR222] Ukil A, Marin L, Jara AJ (2023) Priv-Aug-Shap-ECGResNet: privacy preserving shapley-value attributed augmented resnet for practical single-lead electrocardiogram classification. In: ICASSP 2023-2023 IEEE international conference on acoustics, speech and signal processing (ICASSP). IEEE, pp 1–5

[CR223] Vaduganathan M, Mensah GA, Turco JV, Fuster V, Roth GA (2022) The global burden of cardiovascular diseases and risk: a compass for future health. American College of Cardiology Foundation, Washington 10.1016/j.jacc.2022.11.005 36368511

[CR224] Vafaeezadeh M, Behnam H, Hosseinsabet A, Gifani P (2022) Automatic morphological classification of mitral valve diseases in echocardiographic images based on explainable deep learning methods. Int J Comput Assist Radiol Surg 17(2):413–425 34897594 10.1007/s11548-021-02542-7

[CR225] Valvano G, Leo A, Tsaftaris SA (2022) Regularizing disentangled representations with anatomical temporal consistency. In: Biomedical image synthesis and simulation. Elsevier, Amsterdam, pp 325–346

[CR226] Vaulet T, Al-Memar M, Fourie H, Bobdiwala S, Saso S, Pipi M, Stalder C, Bennett P, Timmerman D, Bourne T et al (2022) Gradient boosted trees with individual explanations: an alternative to logistic regression for viability prediction in the first trimester of pregnancy. Comput Methods Programs Biomed 213:106520 34808532 10.1016/j.cmpb.2021.106520 PMC8674730

[CR227] Vazquez B, Fuentes-Pineda G, Garcia F, Borrayo G, Prohias J (2021) Risk markers by sex for in-hospital mortality in patients with acute coronary syndrome: a machine learning approach. Inform Med Unlocked 27:100791

[CR228] Vijayarangan S, Murugesan B, Vignesh R, Preejith S, Joseph J, Sivaprakasam M (2020) Interpreting deep neural networks for single-lead ecg arrhythmia classification. In: 2020 42nd annual international conference of the IEEE engineering in medicine & biology society (EMBC). IEEE, pp 300–303 10.1109/EMBC44109.2020.9176396 33017988

[CR229] Wagner P, Mehari T, Haverkamp W, Strodthoff N (2024) Explaining deep learning for ECG analysis: building blocks for auditing and knowledge discovery. Comput Biol Med 176:108525 10.1016/j.compbiomed.2024.108525 38749322

[CR230] Wall HE, Hassing G-J, Doll R-J, Westen GJ, Cohen AF, Selder JL, Kemme M, Burggraaf J, Gal P (2022) Cardiac age detected by machine learning applied to the surface ecg of healthy subjects: creation of a benchmark. J Electrocardiol 72:49–55 10.1016/j.jelectrocard.2022.03.001 35306294

[CR231] Wang Q et al (2021a) Machine learning-based risk prediction of malignant arrhythmia in hospitalized patients with heart failure. ESC Heart Fail 8(6):5363–5371 34585531 10.1002/ehf2.13627 PMC8712774

[CR232] Wang S, Li J, Sun L, Cai J, Wang S, Zeng L, Sun S (2021b) Application of machine learning to predict the occurrence of arrhythmia after acute myocardial infarction. BMC Med Inform Decis Mak 21:1–14 34724938 10.1186/s12911-021-01667-8 PMC8560220

[CR233] Wang J, Liu X, Wang F, Zheng L, Gao F, Zhang H, Zhang X, Xie W, Wang B (2021c) Automated interpretation of congenital heart disease from multi-view echocardiograms. Med Image Anal 69:101942 33418465 10.1016/j.media.2020.101942

[CR234] Wang Y, Chen W, Tang T, Xie W, Jiang Y, Zhang H, Zhou X, Yuan K (2022a) Cardiac segmentation method based on domain knowledge. Ultrason Imaging 44(2–3):105–117 35574925 10.1177/01617346221099435

[CR235] Wang X, Qi M, Dong C, Zhang H, Yang Y, Zhao H (2022b) Accurately identifying coronary atherosclerotic heart disease through merged beats of electrocardiogram. In: 2022 IEEE international conference on bioinformatics and biomedicine (BIBM). IEEE, pp 1249–1254

[CR236] Wang J, Xie W, Cheng M, Wu Q, Wang F, Li P, Fan B, Zhang X, Wang B, Liu X (2022c) Assessment of transcatheter or surgical closure of atrial septal defect using interpretable deep keypoint stadiometry. Research 2022:9790653 36340508 10.34133/2022/9790653 PMC9620637

[CR237] Wang K, Yan LZ, Li WZ, Jiang C, Wang NN, Zheng Q, Dong NG, Shi JW (2022d) Comparison of four machine learning techniques for prediction of intensive care unit length of stay in heart transplantation patients. Front Cardiovasc Med 9:863642 35800164 10.3389/fcvm.2022.863642 PMC9253610

[CR238] Wesołowski S, Lemmon G, Hernandez EJ, Henrie A, Miller TA, Weyhrauch D, Puchalski MD, Bray BE, Shah RU, Deshmukh VG et al (2022) An explainable artificial intelligence approach for predicting cardiovascular outcomes using electronic health records. PLoS Digit Health 1(1):0000004 10.1371/journal.pdig.0000004 PMC8975108 35373216

[CR239] Wickramasinghe NL, Athif M (2022) Multi-label classification of reduced-lead ecgs using an interpretable deep convolutional neural network. Physiol Meas 43(6):064002 10.1088/1361-6579/ac73d5 35617943

[CR240] Wong XY, Ang YK, Li K, Chin YH, Lam SSW, Tan KBK, Chua MCH, Ong MEH, Liu N, Pourghaderi AR et al (2022) Development and validation of the sarica score to predict survival after return of spontaneous circulation in out of hospital cardiac arrest using an interpretable machine learning framework. Resuscitation 170:126–133 34843878 10.1016/j.resuscitation.2021.11.029

[CR241] Wongvibulsin S, Wu KC, Zeger SL et al (2020) Improving clinical translation of machine learning approaches through clinician-tailored visual displays of black box algorithms: development and validation. JMIR Med Inform 8(6):15791 10.2196/15791 PMC7312245 32515746

[CR242] Wouters PC, Leur RR, Vessies MB, Stipdonk AM, Ghossein MA, Hassink RJ, Doevendans PA, Harst P, Maass AH, Prinzen FW et al (2023) Electrocardiogram-based deep learning improves outcome prediction following cardiac resynchronization therapy. Eur Heart J 44(8):680–692 36342291 10.1093/eurheartj/ehac617 PMC9940988

[CR243] Wu Z-W, Zheng J-L, Kuang L, Yan H (2023) Machine learning algorithms to automate differentiating cardiac amyloidosis from hypertrophic cardiomyopathy. Int J Cardiovasc Imaging 39(2):339–348 36260236 10.1007/s10554-022-02738-1

[CR244] Wu C, Zhang H, Chen J, Gao Z, Zhang P, Muhammad K, Del Ser J (2022) Vessel-Gan: angiographic reconstructions from myocardial ct perfusion with explainable generative adversarial networks. Futur Gener Comput Syst 130:128–139

[CR245] Wu Z, Li Y, Xu Z, Liu H, Liu K, Qiu P, Chen T, Lu X (2023) Prediction of preoperative in-hospital mortality rate in patients with acute aortic dissection by machine learning: a two-centre, retrospective cohort study. BMJ Open 13(4):066782 10.1136/bmjopen-2022-066782 PMC10083797 37012019

[CR246] Xiao R, Ding C, Hu X, Clifford GD, Wright DW, Shah AJ, Al-Zaiti S, Zègre-Hemsey JK (2023) Integrating multimodal information in machine learning for classifying acute myocardial infarction. Physiol Meas 44(4):044002 10.1088/1361-6579/acc77f PMC10111877 36963114

[CR247] Xing J, Ghadimi S, Abdi M, Bilchick KC, Epstein FH, Zhang M (2021) Deep networks to automatically detect late-activating regions of the heart. In: 2021 IEEE 18th international symposium on biomedical imaging (ISBI). IEEE, pp 1902–1906

[CR248] Yalcin O, Fan X, Liu S (2021) Evaluating the correctness of explainable ai algorithms for classification. arXiv preprint. arXiv:2105.09740

[CR249] Yang H, Shan C, Kolen AF, With PH (2022) Weakly-supervised learning for catheter segmentation in 3d frustum ultrasound. Comput Med Imaging Graph 96:102037 35121377 10.1016/j.compmedimag.2022.102037

[CR250] Ye X, Huang Y, Lu Q (2021) Explainable prediction of cardiac arrhythmia using machine learning. In: 2021 14th International Congress on Image and Signal Processing, BioMedical Engineering and Informatics (CISP-BMEI), pp. 1–5. IEEE

[CR251] Yin C, Zhao R, Qian B, Lv X, Zhang P (2019) Domain knowledge guided deep learning with electronic health records. In: 2019 IEEE International conference on data mining (ICDM). IEEE, pp 738–747

[CR252] Yoo J, Jun TJ, Kim Y-H (2021) xECGNet: fine-tuning attention map within convolutional neural network to improve detection and explainability of concurrent cardiac arrhythmias. Comput Methods Programs Biomed 208:106281 34333207 10.1016/j.cmpb.2021.106281

[CR253] Yue Y, Zhu X (2023) Automated coronary artery disease detection using deep learning on ECG datasets. In: Proceedings of the 2023 3rd International conference on bioinformatics and intelligent computing, pp 242–245

[CR254] Zeng X, Hu Y, Shu L, Li J, Duan H, Shu Q, Li H (2021) Explainable machine-learning predictions for complications after pediatric congenital heart surgery. Sci Rep 11(1):17244 34446783 10.1038/s41598-021-96721-w PMC8390484

[CR255] Zeng Z, Wang Q, Yu Y, Zhang Y, Chen Q, Lou W, Wang Y, Yan L, Cheng Z, Xu L et al (2022) Assessing electrocardiogram changes after ischemic stroke with artificial intelligence. PLoS ONE 17(12):0279706 10.1371/journal.pone.0279706 PMC9794063 36574427

[CR256] Zeng Z, Tian X, Li L, Diao Y, Zhang T (2024) An interpretable machine learning model to predict off-pump coronary artery bypass grafting-associated acute kidney injury. Adv Clin Exp Med 33(5):473–481 37593773 10.17219/acem/169609

[CR259] Zhang X, Qian B, Li X, Wei J, Zheng Y, Song L, Zheng Q (2019a) An interpretable fast model for predicting the risk of heart failure. In: Proceedings of the 2019 SIAM international conference on data mining. SIAM, Philadelphia, pp 576–584

[CR260] Zhang X, Qian B, Li Y, Yin C, Wang X, Zheng Q (2019b) KnowRisk: an interpretable knowledge-guided model for disease risk prediction. In: 2019 IEEE International conference on data mining (ICDM). IEEE, pp 1492–1497

[CR257] Zhang O, Ding C, Pereira T, Xiao R, Gadhoumi K, Meisel K, Lee RJ, Chen Y, Hu X (2021a) Explainability metrics of deep convolutional networks for photoplethysmography quality assessment. IEEE Access 9:29736–29745 33747683 10.1109/access.2021.3054613 PMC7978398

[CR261] Zhang D, Yang S, Yuan X, Zhang P (2021b) Interpretable deep learning for automatic diagnosis of 12-lead electrocardiogram. iScience 24(4):102373 33981967 10.1016/j.isci.2021.102373 PMC8082080

[CR258] Zhang P, Ma C, Song F, Sun Y, Feng Y, He Y, Zhang T, Zhang G (2023) D2AFNet: a dual-domain attention cascade network for accurate and interpretable atrial fibrillation detection. Biomed Signal Process Control 82:104615

[CR262] Zhao S, Diao X, Xia Y, Huo Y, Cui M, Wang Y, Yuan J, Zhao W (2023) Automated ICD coding for coronary heart diseases by a deep learning method. Heliyon 9(3):e14037 36938427 10.1016/j.heliyon.2023.e14037 PMC10018467

[CR263] Zhou D, Qiu H, Wang L, Shen M (2023) Risk prediction of heart failure in patients with ischemic heart disease using network analytics and stacking ensemble learning. BMC Med Inform Decis Mak 23(1):99 37221512 10.1186/s12911-023-02196-2 PMC10207812

