# Supplementary material for: A review of evaluation approaches for explainable AI with applications in cardiology
Source: Artif Intell Rev. 2024 Aug 9;57(9):240. doi: 10.1007/s10462-024-10852-w (PMC11315784; doi:10.1007/s10462-024-10852-w)
Supplement: Supplementary file 1 — Supplementary file1 (DOCX 14 KB) [file 10462_2024_10852_MOESM1_ESM.docx]

Table S1: The query used to search in the literature. This is an example used in Web of Science

| **Term** | **Query** |
| --- | --- |
| Heart | ((AB=(Cardiac) OR AB=(heart)) |
| Data | (AB=(ECG) OR AB=(electrocardiogram) OR AB=(electrocardiographic) OR AB=(electrocardiography) OR AB=(imaging) OR AB=(image) OR AB=(CMR) OR AB=(MRI) OR AB=(X-ray) OR AB= (Computed tomography) OR AB = (ultrasound) OR AB = (echocardiograms) OR AB = (Positron emission tomography) OR AB = (nuclear medicine) OR AB= (clinical data) OR AB= (health records)) |
| Model | (AB= (machine learning) OR AB= (deep learning) OR AB= (Deep neural networks) OR AB= (DNN) OR AB = (Convolutional neural network) OR AB=(CNN) OR AB=(Artificial Intelligence)) |
| Explainable AI | (AB=(XAI) OR AB=(explanation) OR AB=(explainability) OR AB=(interpretability) OR AB=(interpretable) OR AB=(explainable) OR AB = (Partial Dependence Plots) OR AB=(Accumulated Local Effects) OR AB=(eli5) OR AB=(Activation maps) OR AB=(RxNCM) OR AB=(NNKX) OR AB = (SHAPley Additive exPlanations) OR AB=(SHAP) OR AB= (Deep SHAP) OR AB = (Local Interpretable Model-Agnostic Explanations) OR AB=(LIME) OR AB=( Layer-wise Relevance Propagation) OR AB=(LRP) OR AB= (Guided backpropagation) OR AB=(xAI-GAN) OR AB=(DeepLIFT) OR AB =( Seq2Seq-Vis) OR AB=(SmoothGrad) OR AB= (Saliency Maps) OR AB=(DeepTaylor) OR AB=( DeConvNet) OR AB= (Pattern attribution) OR AB = (Integrated gradients) OR AB= (Gradient weighted class activation mapping) OR AB=(Grad-CAM) OR AB=(Grad-CAM++))) |
